# Dual seasonal pattern for hemorrhagic fever with renal syndrome and its potential determinants in China

**DOI:** 10.1016/j.scitotenv.2022.160339

**Published:** 2022-11-22

**Authors:** Chen-Long Lv, Yao Tian, Yan Qiu, Qiang Xu, Jin-Jin Chen, Bao-Gui Jiang, Zhong-Jie Li, Li-Ping Wang, Simon I. Hay, Wei Liu, Li-Qun Fang

**Affiliations:** a State Key Laboratory of Pathogen and Biosecurity, Beijing Institute of Microbiology and Epidemiology, Beijing, China; b Beijing Haidian District Center for Disease Control and Prevention, Beijing, P. R. China; c Division of Infectious Disease, Key Laboratory of Surveillance and Early-Warning on Infectious Diseases, Chinese Center for Disease Control and Prevention, Beijing, China; d Institute for Health Metrics and Evaluation, University of Washington, USA; e Department of Health Metrics Sciences, School of Medicine, University of Washington, USA

**Keywords:** HFRS, seasonality, factors, flood, GAMM, DLNM

## Abstract

Hemorrhagic fever with renal syndrome (HFRS) continued to affect human health across Eurasia, which complicated by climate change has posed a challenge for the disease prevention measures. Nation-wide surveillance data of HFRS cases were collected during 2008–2020.The seasonality and epidemiological features were presented by combining the HFRS incidence and the endemic types data. Factors potentially involved in affecting incidence and shaping disease seasonality were investigated by generalized additive mixed model, distributed lag nonlinear model and multivariate meta-analysis. A total of 76 cities that reported totally 111,054 cases were analyzed. Three endemic types were determined, among them the Type I cities (Hantaan virus-dominant) were related to higher incidence level, showing one spike every year in Autumn-Winter season; Type II (Seoul virus-dominant) cities were related to lower incidence, showing one spike in Spring, while Type III (Hantaan/Seoul-mixed type) showed dual peaks with incidence lying between. Persistently heavy rainfall had significantly negative influence on HFRS incidence in Hantaan virus-dominant endemic area, while a significantly opposite effect was identified when continuously heavy rainfall induced floods, where temperature and relative humidity affected HFRS incidence via an approximately parabolic or linear manner, however few or no such effects was shown in Seoul virus-dominant endemic areas, which was more vulnerable to temperature variation. Dual seasonal pattern of HFRS was depended on the dominant genotypes of hantavirus, and impact of climate on HFRS was greater in Hantaan virus-dominant endemic areas, than in Seoul virus-dominant areas.

## Graphical Abstract

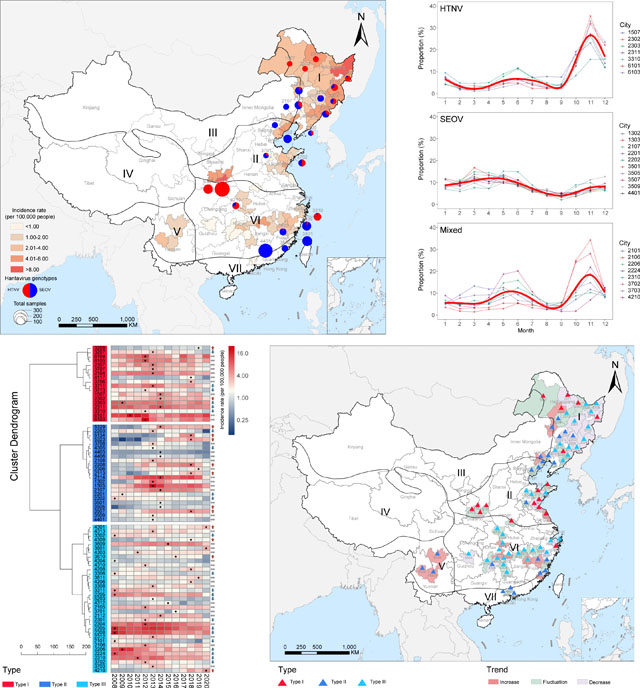

## 1. Introduction

Hemorrhagic fever with renal syndrome (HFRS), a natural focal disease with rodents as the main natural host and infectious source, is characterized by fever, bleeding, hypotension, headache, back pain, abdominal pain and acute renal insufficiency with a case fatality rate of 1%–15% (Avsic-Zupanc et al., 2019; Cao et al., 2020). As many as 150,000 HFRS cases were reported globally per year (Jonsson et al., 2010), mainly caused by Hantaan virus (HTNV) in Asia, Puumala virus (PUUV) and Dobrava-Belgrade virus (DOBV) in Europe, and Seoul virus (SEOV) worldwide. (Wang et al., 2020) The majority of HFRS cases are reported in Asia, specifically China, resulting from infection with HTNV (carried by wild rodents, e.g., *Apodemus agrarius* and *Apodemus peninsulae*) and SEOV (carried by commensal rodents, e.g., *Rattus norvegicus* and *Mus musculus*), with >1.5 million cases between 1950 and 2007 (Zhang et al., 2010). Cases of HTNV are also reported in Russia and Korea, while DOBV carried by *Apodemus flavicollis* and PUUV carried by *Myodes glareolus* are most frequently reported in Europe, particularly in Scandinavia and western Russia (Avsic-Zupanc et al., 2019; Heyman et al., 2011) As the most endemic country, China has reported HFRS cases in all the 31 provinces (autonomous regions and municipalities) (Sun et al., 2021). Although the incidence of HFRS has been remarkably decreased via the implementation of HFRS vaccination since 2003 (Brocato and Hooper, 2019), there has been a rebounding incidence since 2009 (Xiang et al., 2018; Bai et al., 2019), which remains a serious public health problem in China.

Human outbreaks occur annually in China, which had been primarily documented in the high-endemic regions in northeastern and western China (Ma et al., 2014; Fang et al., 2006; Shang et al., 2020). However, there is variability in the geographic locations, the seasonal timing, as well as the number and magnitude of human epidemic or outbreaks that occur each year (Zou and Sun, 2020; Wang et al., 2010). Currently, spatial analyses of HFRS at the country scale are rare, and the only one study recently reported was limited to the calculation of incidence rate (Sun and Zou, 2018). Several studies have explored the main drivers of spatial and temporal patterns of HFRS at the province level, revealing strong correlations between HFRS risk and suitable environments for rodents, including grass and shrub cover, as well as forested land fragmented by agricultural or shrub cover (Fang et al., 2009; She et al., 2021). Non-irrigated agricultural land cover has also been found to be associated with HFRS incidence in Jilin (Northeast region) and Hunan (Central China region) (Wu et al., 2014; Xiao et al., 2018). The recent studies had also intensively related the effects of climatic factors (mainly rainfall, temperature and humidity) on the incidence of HFRS in China, primarily performed in the provinces of Inner Mongolia, Heilongjiang and Liaoning in the northeastern China; Shandong and Jiangsu in the eastern China; Anhui and Hunan in central China (Bi et al., 2005; Zhang et al., 2010b; Li et al., 2013; Xiao et al., 2016; Zhang et al., 2019). However, high discrepancies remained as to the direction and extent of the effect, which might arise from various climate zones and ecological characteristics of the study regions. Heterogeneity in the methodology existed across studies and is likely to have biased the estimates, hence the results were rarely replicated. Most critically, the effects of environment and climate on HFRS with no due consideration of the two separate viruses can be misleading and erroneous at worst. Climatic events, especially heavy rains and flooding following periods of drought, have precipitated rodent-borne infectious disease outbreaks, which extreme weathers, however have been rarely studied for their effects on HFRS.

In the current study, we compiled a national database of reported human cases with HFRS, in combination with the literature review data, GenBank database, and the yearly routine etiological surveillance on the genotyping of hantavirus, to estimate the timing of the HFRS seasonality, comparing and contrasting the seasonality of HFRS across HTNV- and SEOV-dominant endemic areas. We further identified the determinants associated with regional heterogeneity of HFRS incidence and the heterogenic effects of climatic indicators on HFRS incidence. The current study might increase understanding of the interactions between environmental, demographic, social, virological, and other factors that might drive these patterns of disease, which is crucial to mitigating disease risk.

## 2. Material and methods

### 2.1. Data collection and management

In our database, we included individual data of all laboratory-confirmed and clinically confirmed cases during 2008–2020 that were collected by the China Information System for Disease Control and Prevention (CISDCP), a national surveillance system serving the general population of an entire country. All data were collected with no age or patient-based restrictions. To ensure the highest quality data included to reflect the seasonal characteristics, we used only the cities with cumulative cases >500 during the study period (Table S1).

Data on 3 social-demographic, 3 meteorological and 3 environmental factors potentially associated with HFRS incidence were collected and processed at the city level (Table S2–S3). Among them, city-level social-demographic data including annual population density, annual proportion of urban population, and annual gross domestic product (GDP) per capita were provided by National and Local Bureau of Statistics. The raster-type map lays of monthly meteorological indicators with a spatial resolution of 5 km, including cumulative rainfall, average temperature, and average relative humidity (RH), were extrapolated by the inverse distance weighted interpolation technique using 613 weather surveillance stations in China, which were obtained from Chinese National Meteorological Information Center (http://data.cma.cn/). The raster-type map lays of environmental data included annual average normalized difference vegetation index (NDVI) with a spatial resolution of 1 km that comprehensively reflects the vegetation growth in an area (Ji et al., 2020) and 2010 elevation with a spatial resolution of 1 km which were collected from Resource and Environment Science and Data Center (https://www.resdc.cn/), as well as land cover with 8 categories with a spatial resolution of 0.3 km which were collected from European Space Agency (https://www.esa.int). All these raster-type map lays were overlapped on the city-level vector digital map of China and these covariates of each city included in this study were calculated by using the zonal statistical calculation technique. The calculation and operation on raster type data were performed by using ArcGIS 10.7 (Environmental Systems Research Institute Inc., Redlands, CA, USA).

Extreme weather conditions were quantitively estimated by the Standardized Precipitation Index (SPI), which has been applied in the drought and flood surveillance by the National Climate Center of China Meteorological Administration (China Meteorological Administration, 2017). Based on the meteorological data collected at the city level, the monthly SPIs were calculated for each city included in this study from 2008 to 2020 (Supplementary Material and Methods and Table S4). Drought and flood were defined as SPI <−1.5 and >1.5, respectively as previously described (Shao and Kam, 2020). The urbanization at the city level was assessed by using the composite urbanization index that was estimated based on proportion of urban population, GDP per capita and built-up area per capita with entropy weight method (detailed in Supplementary Material and Methods and Table S5) (Shannon, 1948).

### 2.2. Determination of endemic types based on hantavirus serotypes

Because no information about serogroups or serotypes was reported in the CISDCP, we assembled an additional database on the hantavirus serotypes determined from either rodents or humans via literature review in Google, Google Scholar, PubMed; by data extraction in GenBank and the national etiological surveillance data of hantavirus in humans and rodents in China (https://www.chinacdc.cn/). Briefly, we searched public databases using general search terms such as “rodent”, “Hantavirus”, and “China”. According to residential address or sampling address at the city level, the hantavirus serotypes were geo-referenced and related to the geographic location of the reported cases (detailed in Supplementary Material and Methods). Out of the 76 selected cities, 25 reported over 40 patients or rodents with determined hantavirus genotypes, which were used for the definition of endemic type. Briefly, three endemic types, Type I, Type II or Type III, that corresponded to the HTNV-dominant (with HTNV accounting for ≥80% of all genotyped cases), SEOV-dominant (with SEOV accounting for ≥80% of all genotyped cases), and mixed type (both HTNV and SEOV mixed with comparable proportion), were defined respectively. Seasonal pattern was determined for these three types of HFRS endemic cities, which was applied in the hierarchical cluster analysis to classify all the remaining 51 cities into three types (I–III). Briefly, the proportions of HFRS cases in each month within a year at the city level were used as input, based on which the Ward’s minimum-variance distance was used to define three clusters which corresponded to types I, II, and III.

### 2.3. Statistics and modeling

The epidemiological features of patients from these three types of endemic regions were described over time and by locations. The medians and interquartile ranges (IQRs) were calculated for continuous variables, while the frequencies were calculated for categorical variables. Kruskal-Wallis rank-sum tests were applied to compare continuous variables. Pearson’s Chi-square test or Fisher’s exact test was used for the comparison of categorical variables. The Mann-Kendall trend test was used to examine the trend of HFRS incidence across the study years and the age groups. The generalized additive mixed model (GAMM) was performed to explore the factors associated with the spatial distribution of annual incidence rate of HFRS for types I and II endemic regions, separately. The explanatory variables used in the GAMM model included land cover per capita, elevation, NDVI and urbanization index. Random intercept was included in the models to explain variation due to sampling from different cities. Models were implemented by R package “mgcv” and specified using a Poisson distribution, a log link function, restricted maximum likelihood (REML) and cubic regression splines with knot-based approximations were applied for spatial smoothing (detailed in Supplementary Material and Methods) (Wood, 2006). The patients from the Type III cities were not included in the modeling analyses, due to the potentially inseparable effect between HTNV and SEOV. The association between influencing factors and HFRS incidence was assessed by a piecewise linear Poisson regression to allow different slopes before and after a threshold value (Zhu and Xie, 2020; Li et al., 2021a). The best piecewise regression model was the one in which the threshold value minimized the generalized cross-validation value.

The lag effect of meteorological factors on HFRS incidence was assessed by applying quasi-Poisson regression with distributed lag nonlinear model (DLNM) for types I–III regions, respectively by using R package “dlnm” (Gasparrini et al., 2010). Four meteorological indicators with monthly value data, including average temperature, cumulative rainfall, average RH, and SPI, were calculated for each city and were applied in the model (Supplementary Material and Methods). Considering the immense spatial heterogeneity caused by different exposure ranges of the climatic indicators among cities, we used the original values of temperature (rainfall or RH) for each city as the input of city-specific DLNM, and selected internal nodes at the same percentiles in spline functions for all cities of types I–III, hence the parameter estimations of the percentiles could be obtained finally, which actually corresponded to different absolute temperature (rainfall or RH). Then the city-specific estimation of types I–III for each meteorological indicator (temperature, rainfall and RH) obtained from the DLNM were then combined by corresponding percentiles separately through random-effect multivariate meta-analysis using the REML (Harville, 1977), which was implemented in the R package “mvmeta” (detailed in Supplementary Material and Methods). SPI retained at its original scale because of the largely overlapping numerical ranges for all the cities.

## 3. Results

### 3.1. Determination of three endemic types of HFRS

**Fig. 1.**
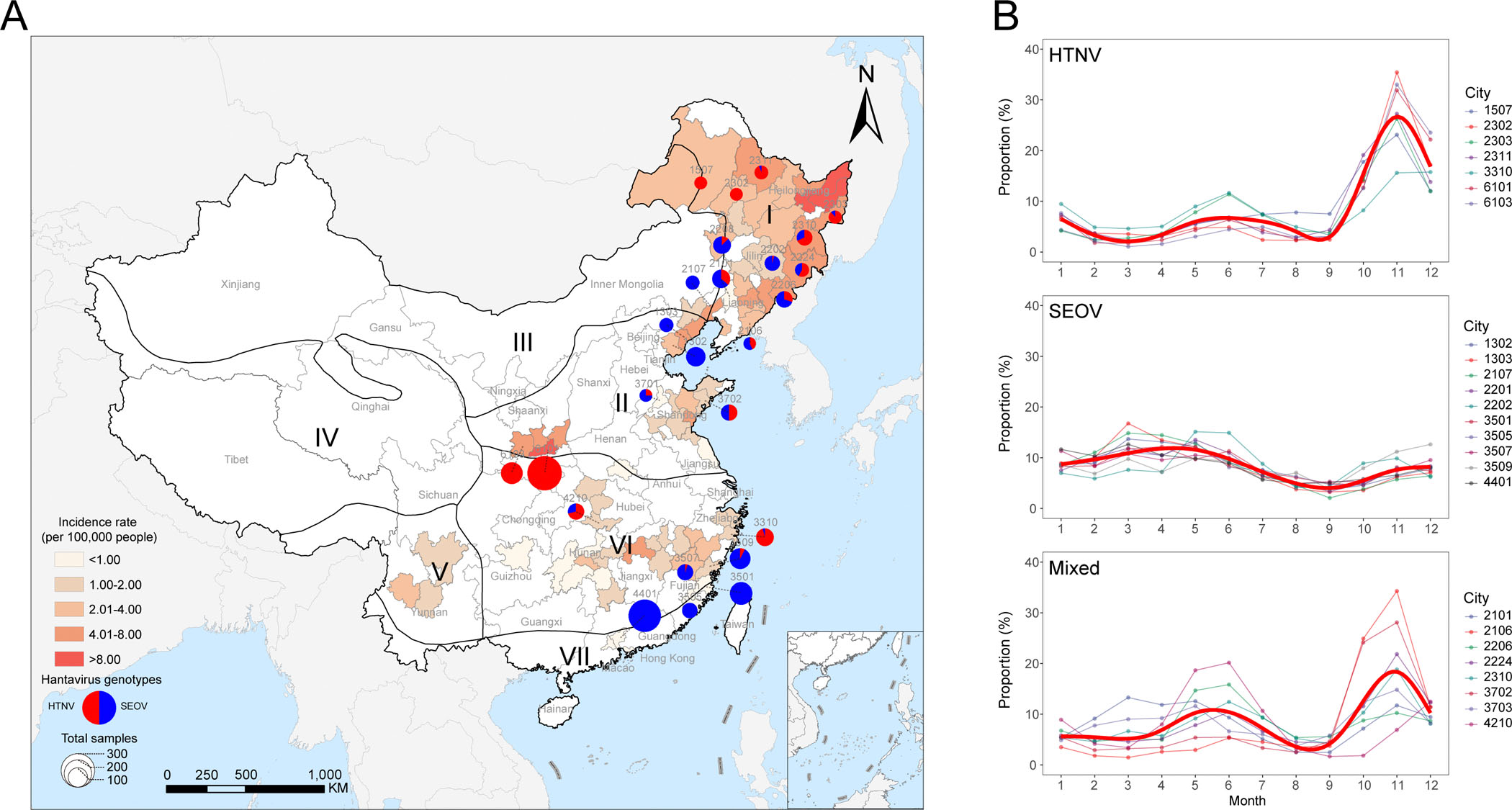
Geographic distribution and seasonality of patients with HFRS in the mainland of China, 2008–2020. (A) The case incidence of HFRS in 76 selected cities (with cumulative number of cases >500) was denoted by background color. The endemic type of 25 selected cities (with total number of genotyped samples >40) was presented by pie chart. The size of pie indicated the total genotyped samples. I = Northeast region, II = North China region, III = Inner Mongolia-Xinjiang region, IV = Qinghai-Tibet region, V = Southwest region, VI = Central China region, and VII = South China region. (B) The seasonality of HFRS case was presented by the average monthly proportion of HFRS cases across 2008–2020 stratified by predominant genotypes. The bold red line indicated the fitting incidence of HFRS. The codes of the selected 25 cities were noted in both panels. HFRS: hemorrhagic fever with renal syndrome.

During 2008–2020, totally 137,263 HFRS cases were reported from 308 cities in all 31 provinces in the mainland of China. Among them 76 cities that had reported over 500 cumulative cases were used for the current analysis. The 76 cities, although mainly located within five ecological regions (Northeast region, North China region, Central China region, Southwest region, and South China region), had reported 80.91% of nation-wide cases (111,054/137,263), showing a spatial clustering. A higher average annual incidence was observed in Northeast and North China regions than those in Southwest, Central China and South China regions (Fig. 1A).

Totally 3 of 76 cities had annualized average incidence rates over 8.00 per 100,000 people, with two located in Heilongjiang of Northeast region and one in Shaanxi provinces of North China region. Another 16 cities had annualized average incidence ranging from 4.01 to 8.00 per 100,000, with most of them (10 cities) were again located in Northeast China, and three in Shaanxi province (Fig. 1A). Among them 25 cities that had produced over 40 human cases or rodents with determined hantavirus types during 2008–2020 were categorized into HTNV-type (7 cities), SEOV-type (10 cities) and mixed-type (8 cities), respectively. The cities were evenly located at Northeast and Inner Mongolia-Xinjiang regions (4 Type I, 3 Type II and 5 Type III cities), North China region (2 Type I, 2 Type II and 2 Type III cities), and Central and South China regions (1 Type I, 5 Type II and 1 Type III cities), thus indicating a good geographic representativeness of the cities (Fig. 1A). The three types of endemic regions showed distinct seasonality, the seasonal timing of HTNV was situated in October to December, during the Autumn-Winter season, SEOV was situated in March to May, during the Spring season, and both seasonal timing was observed for the mixed type cities (Fig. 1B).

### 3.2. Demographic and environmental characteristics compared across endemic types

**Fig. 2.**
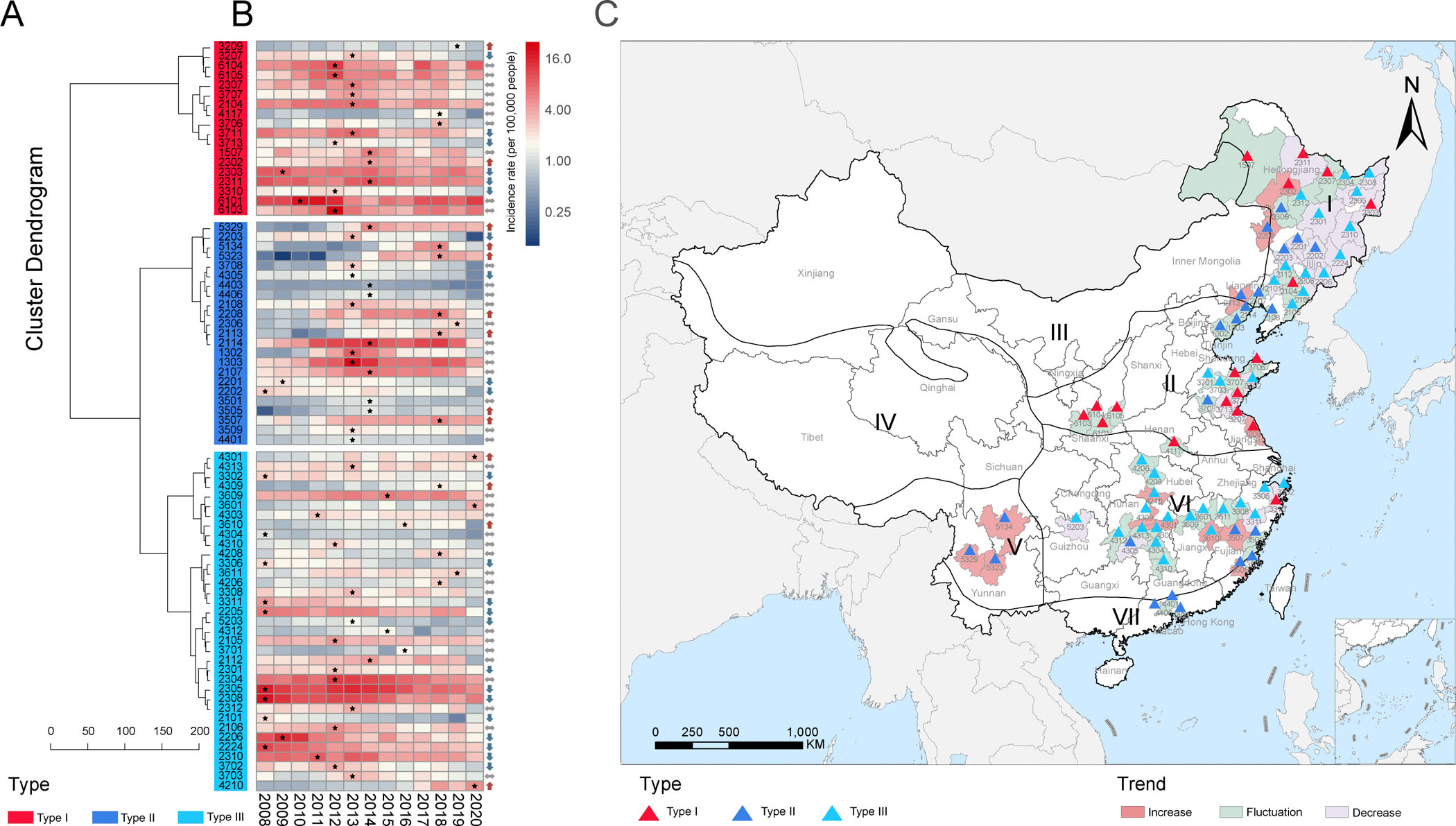
Dynamics and seasonality of HFRS in endemic areas with different types based on hierarchical cluster analysis combined with dominant hantavirus genotypes. (A) The cluster dendrogram of the remaining cities after excluding those that had been classified by dominant hantavirus genotypes. (B) Annually changing trend of HFRS incidence in each city. The asterisk and arrow represent the peak and changing direction of annual incidence rates in the city, respectively. (C) Spatial distribution of endemic areas with different types based on hierarchical cluster analysis combined with dominant hantavirus genotypes. The colored triangle represents the corresponding type of HFRS seasonality for each city. The white background indicates the un-selected areas in this study, and the other three colored backgrounds indicate the annual changing trends corresponding to the selected cities. The name of each province, municipality, autonomous region and the code of each selected city are noted. I = Northeast region, II = North China region, III = Inner Mongolia-Xinjiang region, IV = Qinghai-Tibet region, V = Southwest region, VI = Central China region, and VII = South China region. HFRS: hemorrhagic fever with renal syndrome.

Combined with the hierarchical cluster analysis on the monthly incidence for the remaining 51 cities, all of the 76 selected cities were classified into three endemic types, which comprised Type I of HTNV-dominant (18 cities), Type II of SEOV-dominant (23 cities), and Mixed endemic areas (35 cities) (Fig. 2A and Table S6). The highest incidence was observed for Type I region (3.487 per 100 thousand persons), followed by Type III (2.082/100,000) and Type II (1.602/100,000) (Table 1). The case fatality rate (CFR) also differed among three-type endemic areas, with significantly higher CFR related to types I (0.842%) and III (0.887%) than Type II (0.222%) (p <0.001). A higher richness of endemic types was determined from North China and Northeast China regions than in other regions (Type II mainly in South and Central China regions, and Type I mainly in Northeast and North China regions) (Fig. 2C).

The mean age of all the patients was 46.2 (standard deviation, 15.5), with slightly older age observed in Type I (46.5±16.0) and Type III (46.5±14.9) cities, than in Type II (45.0±15.7) (p <0.05). An age dependent increase in case incidence was observed, with the highest incidence seen in the >49 years group (p <0.05). The same age trend was likewise observed across the three endemic types and across the studied years, except for an unexpectedly higher incidence in the 0–9 years group than the 10–19 years group for types II and III during 2013–2015 and 2015–2017, respectively (Fig. S1). A higher incidence was observed in male than in female for each type of endemic cities. Overall incidence decreased from 2.084/100,000 in 2008 to 1.678/100,000 in 2020, however, there was a significant 5-year periodicity, with two peak years observed in 2012 and 2017 for Type I, in 2013 and in 2018 for Type II cities, respectively (Fig. S2). Comparable proportion of drought months and flood months were observed for the three-type of cities (both p >0.05). Except for elevation, population density and the areas of wetland per capita, all the 8 other environmental indicators, including NDVI, areas per capita of rainfed cropland, irrigated cropland, broad-leaved tree, needle-leaved tree, shrubland, grassland, and built-up land, differed significantly among the three-type endemic areas (Table S7).

### 3.3. Heterogenous association with HFRS incidence across three endemic types

**Fig. 3.**
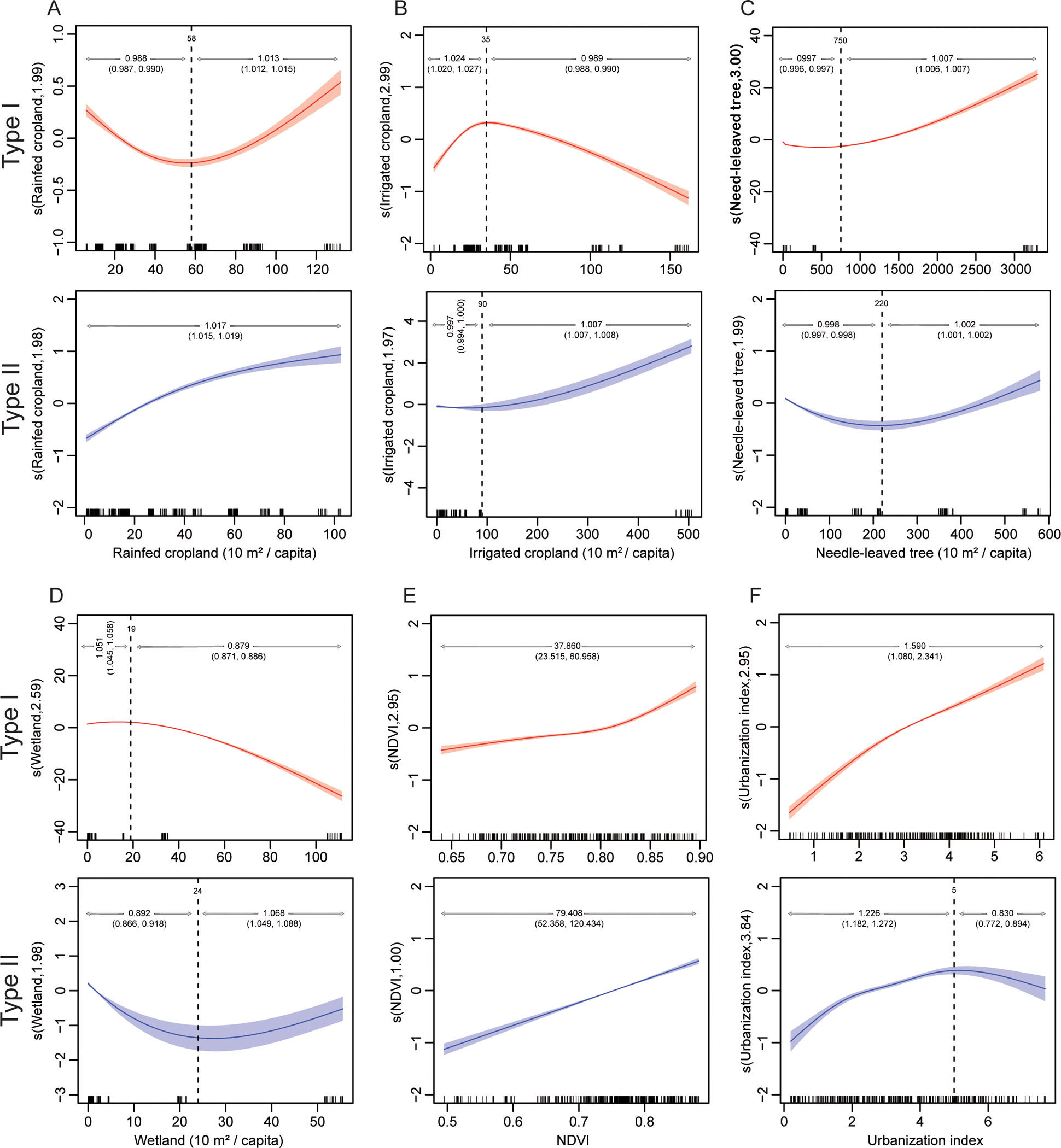
Pooled exposure-response curves between the common habitat factors and the annualized incidence rates of HFRS for types I and II endemic cities based on GAMM. (A) Rainfed cropland. (B) Irrigated cropland. (C) Needle-leaved tree. (D) Wetland. (E) NDVI. (F) Urbanization index. The x-axis indicates the observed values of the habitat factors, while the y-axis indicates the contribution of the smooth term to the fitted values with the EDF in parentheses. The relative risks are marked above the curves with their 95%CIs in parentheses, which were calculated based on the piecewise linear Poisson regression. The associations of elevation with Type I, and shrubland and grassland with Type II are shown in Fig. S3. Broad-leaved tree, shrubland and grassland were excluded because of their collinearity with NDVI, needle-leaved tree and wetland for Type I, respectively, while broad-leaved tree and elevation were excluded because of their collinearity with shrubland and needle-leaved tree for Type II, respectively. HFRS: hemorrhagic fever with renal syndrome. GAMM: generalized additive mixed model. CI: confidence interval. NDVI: normalized difference vegetation index. EDF: effective degrees of freedom of the smooth function term (EDF >1 indicates nonlinear association).

Based on the GAMM at city level, seven variables were significantly associated with HFRS annual incidence in Type I cities, and eight were significant for Type II, with six of them significant for both types of endemic cities, i.e., rainfed cropland, irrigated cropland, needle-leaved tree, wetland, NDVI and urbanization index (Table S8). Except for NDVI, five other variables showed nonlinear effect for their association with HFRS incidence which differed between types I and II (Fig. 3). For example, the areas of rainfed cropland or needle-leaved tree per capita affected the two types of endemic cities through the same way, featuring that the incidence first decreased and then increased, except rainfed cropland for Type II (Fig. 3A and C). The areas of irrigated cropland or wetland per capita had a bidirectional and nonlinear effect on the risk of HFRS incidence for Type I cities, while with monotonous increase for the former and early decrease and late increase for the latter in the Type II cities, respectively (Fig. 3B and D). NDVI affected the HFRS incidence in a consistent way for types I and II (Fig. 3E). The urbanization index showed different effect on HFRS, which increased by 59% for Type I, and early increased by 23% and late decreased by 17% for Type II, by every one-unit change, respectively (Fig. 3F). In addition, the elevation was significantly correlated with HFRS incidence only for Type I and in a non-linear manner, while the shrubland and grassland per capita had both shown a non-linear association with the HFRS risk only for Type II (Fig. S3).

At the city level, increase trend of incidence was observed in 30% of the Type II cities, more than those in Type I (11%) and Type III (11%) (Fig. 2B). Compared with cities with no change or decreased incidence, those with increased incidence were associated with more areas of rainfed cropland, grassland, wetland and built-up land per capita observed in Type I cities, higher elevation, more irrigated cropland, broad-leaved tree, needle-leaved tree and grassland per capita observed in Type II cities, and more rainfed cropland and wetland per capita observed in Type III cities, respectively (all p <0.05) (Table S9).

### 3.4. Lag effects of climatic factors on HFRS incidence

**Fig. 4.**
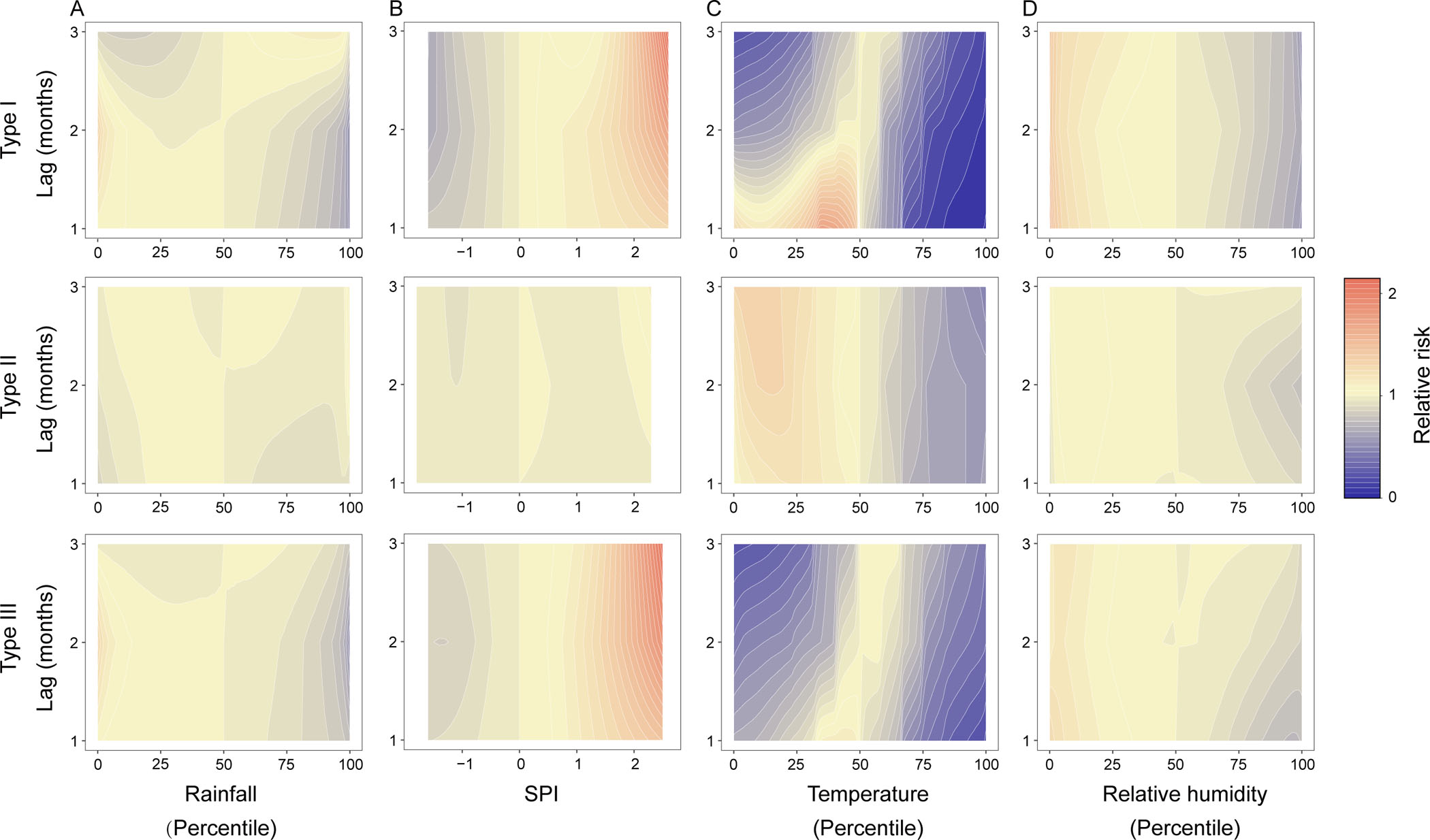
Contour plot of the association between the meteorological factors and the monthly incidence rates of HFRS during different lag periods for the three types of endemic cities based on DLNM. The parameter estimations of three meteorological factors including monthly cumulative rainfall (A), monthly average temperature (C), and monthly average RH (D) were all transformed into percentile scale in analysis with the references at their respective medians, while the SPI (B) retained its original scale with the reference at 0. The deeper the shade of red, the greater the increase in relative risk of HFRS compared with the references. The deeper the shade of blue, the greater the decrease in relative risk of HFRS compared with the references. DLNM: distributed lag nonlinear model. HFRS: hemorrhagic fever with renal syndrome. SPI: standardized precipitation index.

In the Type I cities, a bidirectional effect from the level of monthly rainfall was observed. As rainfall increased from 45.738 to 90.581 mm, the risk of HFRS decreased by 13% with a delay of 1–2 months (Table 2, Fig. 4A and Table S10). However, when rainfall reached the flood level, a positive effect on the disease incidence was observed, with the risk elevated by 16%–23%, similarly with 1–2 months lag (Figs. 4B and Fig. S4). The precipitating effect from flood was slightly enhanced as the longer lag duration, but the impact of high rainfall would gradually disappear. By contrast, less rainfall and to the level of droughts were significantly associated with reduced incidence risk by 14% and 32%, separately with a lag of 3 months. The opposite effect of persistently heavy rainfall and flood on HFRS incidence was also identified in the Type III cities with a delay of 1–2 months (Figs. 4 and S6).

The impact of temperature on HFRS incidence followed parabolic and linear pattern for types I and II (Figs. S4–S5). For Type I cities, when temperature fell below the median level, its effects on the incidence of HFRS changed from positive association to negative association (Fig. 4C). In a similar way for Type II, lower temperature was related to significantly increased risk of HFRS with a lag of 2–3 months. When temperature was elevated above the median level, consistent effect on incidence was observed for two types, featuring that the risk of the disease decreased by 48%–85% for Type I and by 29%–35% for Type II with temperature increasing from 14.175 to 21.183°C and from 15.193 to 20.912°C, respectively (Table S10). RH exerted similar effect, which was negatively correlated to the incidence rates with a delay of 1–3 months for Type I, while a weakly negative effect of high RH on HFRS was observed merely at a lag of 2 months in Type II cities (Figs. 4D and S5). In general, the association between HFRS incidence and temperature or RH in Type III cities was closer to that in Type I cities (Table 2 and Fig. S6).

## 4. Discussion

HTNV and SEOV, two predominant causative pathogens of HFRS in China, are usually harbored by wild rodents, e.g., *Apodemus agrarius* and *Apodemus peninsulae*, and by commensal rodents, e.g., *Rattus norvegicus* and *Mus musculus*, respectively. Despite of previous evidence that HNTV-type and SEOV-type patients tend to vary for the timing of infection, we provided the first evidence highlighting the difference in the demographic, climatic conditions, ecological environment, social economy factors for the human infection stratified by two distinct serotypes. The foremost difference was observed for the epidemic timing, which occurred in Autumn-Winter and Spring seasons for HTNV and SEOV, respectively. This distinct seasonal spikes in human infection might coincide with annual or semiannual synchronous birth pulses of two rodent species. Moreover, we revealed a five-year periodicity in the peaking occurrence for both types of endemic areas. This multiyear periodicity in the incidence of HFRS could arise from a buildup and waning of herd immunity in the reservoir host, with reintroduction of virus via immigration, recrudescence, or viral persistence. A spatial-temporal analysis of human HFRS had shown a significantly more increased HFRS incidence in the Type II regions related to SEOV, which were mainly located in Central, Southwest and South China regions, e.g., Fujian, Jiangxi, Hunan, Hubei, Yunnan and Sichuan provinces. All these distinctions highlight that the environmental and ecological determinants and their effect differed between two types of regions. This had necessitated a comprehensive understanding of human hantavirus infection in the context of the genotyping data.

Further by applying two separate models to distinguish between two types of regions, we had yielded discrepant or even contrasting effect from the risk factors on disease risk. Incidence of Type I was more prone to be affected by meteorological changes than the Type II. We additionally displayed opposite effect of high amount of rainfall and flood on the incidence of HFRS in the Type I cities (Fig. 4A and B). Adequate precipitation can contribute to the growth of vegetation serving as food for rodent hosts (Xiao et al., 2013; Tian et al., 2017b), nevertheless, excessive rainfall will lead to the inundation of the rodent cave, the drowning of the rodent pups and even destructing the entire habitat (Brown and Ernest, 2002; Li et al., 2021b). When combined with the reduced outdoor activity of human beings due to frequent rainfall, an interactive effect was elicited which further reduced the incidence (Fang et al., 2010; Bi and Parton, 2003).

As we known, the urban construction will affect the habitats of rodents and result in rats and people cohabitating in cities, which provide more opportunities for human-rodent interaction and increase human infection with SEOV (Himsworth et al., 2013). However, this risk might decrease when people’s standards of living increase. i.e., some cities such as Beijing and Tianjin, the urbanization occurred earlier and also caused a temporary increase in incidence of human infection of SEOV in 2000s (Fang et al., 2009; Tian et al., 2018), HFRS incidence decreased with the completion of urbanization of them. Hence, authorities need to be vigilant in the surveillance of infected rodent populations for those cities with rapid urbanization and more urban construction.

The level urbanization was positively related to increased incidence for Type II regions at initial stage of urbanization (Fig. 3F). It’s been suggested that land conversion led to unprecedented mixing of wild rodent species and humans from previously unconnected biological communities, causing higher pathogen exposure and resulting in the increased incidence in human populations. Of note, the epidemic dynamics of HFRS changed tremendously when continuously heavy rainfall induced floods. The potential promoting effect of flood on rodent-borne diseases had been reported both at home and abroad (Diaz, 2015; Brown and Murray, 2013; Xu et al., 2014; Wang et al., 2017). During flooding, the surviving rodents may massively assemble on the higher terrain where the displaced people are located in a short time (Diaz, 2014). Not only that, the possibility of mutual diffusion of pathogens within populations also increases considerably as a consequence of repeated contact among rodents (Wójcik-Fatla et al., 2013). Meanwhile, overcrowding, poor sanitary and medical conditions, unhealthy diet, decreased immunity and other factors jointly provide a hotbed for the epidemic of HFRS as well. On the other hand, the damaged areas usually leave abundant food source for rodents after floods, leading to the rodent populations recovering rapidly (Brown and Murray, 2013). Therefore, the flood disaster appears to be a risk factor for the transmission of HFRS both in short-term and long-term (Fig. 4B), suggesting that it is necessary to continuously strengthen the prevention of HFRS in the process of disaster recovery especially in HTNV-dominant endemic areas. Regarding the extremely dry weathers, they appeared to have significantly negative effects on the transmission of hantavirus in Type I cities (Table 2 and Fig. 4B), possibly due to the decline of rodent population caused by the low reproductive rate and food shortage under dry conditions (Liu et al., 2013; Tian et al., 2017a). Relatively, *Rattus norvegicus* has a close relationship with humans and does not rely on farm crops, therefore, hantavirus infection associated with it in SEOV-dominant endemic areas may not be significantly affected by droughts (Fig. 4B) (Tian et al., 2017a).

The temperature affects rodent dynamics, viral infectivity as well as human activity (He et al., 2018; Kallio et al., 2006; Tian et al., 2015; Lin et al., 2014). For the Type I cities, the lag impacts of temperature on the incidence of HFRS had shown a nonlinear manner (Fig. 4C) (Tian et al., 2017b; Lin et al., 2014). Studies have suggested that the breeding rate of rodents is highest at temperatures of 10–25°C (Lin et al., 2014; Guan et al., 2009), however, the reproductive of *Apodemus agrarius* can be inhibited (Liu et al., 2013) and the survival time of virus in vitro can be shortened (Hardestam et al., 2007) when the temperature is outside the range. As to Type II, it has been proved that the Winter temperature can affect the food supply and overwintering survival of rodents (Korslund and Steen, 2006; Aars and Ims, 2002), so colder weather may increase the dependence of *Rattus norvegicus* on human living environment, thus contributing to a high risk of HFRS at low temperatures, which indicates that the cold weather in Spring needs additional attention in SEOV-dominant areas (Fig. 4C). In addition, the lag effects of relative humidity on the incidence of HFRS displayed an approximately negatively linear relationship in Type I cities (Fig. 4D), probably due to the more negative influence on the infectivity and stability of hantavirus caused by higher levels of RH (Zhao et al., 2019).

The limitations of this study should also be acknowledged. First, some of the genotyping data were retrieved from literature source, for which the selections of survey settings of rodents and HFRS patients for genotyping of hantavirus were not performed by unified standards and the sampling was likely not random, thus inevitably introducing bias in genotyping results, which could affect the identification of endemic types. Therefore, we first identified the endemic types (types I–III) for 25 cities where over 40 samples were determined for hantavirus genotype, and endemic types of the other 51 cities with few samples (<40) identified for hantavirus genotypes were then classified by using hierarchical cluster analysis, based on the seasonal characteristic of different endemic types identified by the 25 cities, which might have reduced the impact from this potential bias. In addition, some potential factors of HFRS incidence were not included in our modeling analysis due to the lacking of data, e.g., deratization, vaccination program for high-risk population group aged 16–60, public education about HFRS (Brocato and Hooper, 2019; She et al., 2021; Yi et al., 2019), which would inevitably result in a potential bias in the estimation of relative risk for these potential determinants identified in this study, and further systematic epidemiological studies that fully consider these factors are called for.

## 5. Conclusions

In conclusion, our study has disclosed the link between dual seasonal patterns of HFRS incidence with hantavirus genotypes detected from human and rodents by using nation-wide HFRS data, and has quantitated the effects of different-level rainfall on its incidence in HTNV-dominant endemic areas. The identification of seasonal pattern specific to endemic types and five-year periodicity could provide accurate forecasts of HFRS cases and could be adopted as an early warning surveillance for HFRS outbreaks. Our modeling results have filled the crucial gaps of factors associated with the HFRS epidemic, especially climatic indicators and extreme weather conditions, which might help to make targeted measures for prevention and control in different endemic areas.

## Supplementary Material

Supplementary appendix

Appendix A. Supplementary data

Supplementary data to this article can be found online at https://doi.org/10.1016/j.scitotenv.2022.160339.

## Figures and Tables

**Table 1:** Basic characteristics of three types of HFRS endemic cities in the mainland of China, 2008–2020.

	Type I cities (N=18)	Type II cities (N=23)	Type III cities (N=35)	*p* value

Demographic characteristics				
Number of cases	41,937	26,600	42,517	
Incidence rate (per 100,000 people)	3.487	1.602	2.082	**<0.001**
Gender (incidence, per 100,000 people)				**<0.001**
Male	31,398 (5.208)	18,978 (2.370)	31,794 (3.046)	
Female	10,539 (1.805)	7,622 (1.017)	10,723 (1.068)	
Age, years (incidence, per 100,000 people)				**<0.001**
0−9	334 (0.283)	270 (0.178)	307 (0.149)	
10−19	2,237 (1.564)	1,191 (0.660)	1,478 (0.624)	
20−29	4,605 (2.419)	3,266 (1.041)	4,255 (1.272)	
30−39	6,174 (3.406)	4,732 (1.703)	7,180 (2.138)	
40−49	9,662 (4.415)	6,485 (2.419)	10,931 (2.920)	
>49	18,925 (5.658)	10,656 (2.980)	18,366 (3.294)	
Number of deaths	353	59	377	
Case fatality rate (%)	0.842	0.222	0.887	**<0.001**
Meteorological characteristics				
Number of droughts (occurrence months per city, %)^†^				0.833
No	150.556 (96.510)	149.087 (95.569)	148.457 (95.165)	
Yes	5.444 (3.490)	6.913 (4.431)	7.543 (4.835)	
Number of floods (occurrence months per city, %)^†^				0.986
No	143.669 (92.016)	142.913 (91.603)	143.000 (91.806)	
Yes	12.333 (7.984)	13.087 (8.397)	13.000 (8.194)	

† An extreme weather with SPI less than −1.5 is defined as a drought, and with SPI > 1.5 is defined as a flood.

The number of droughts (or floods) counts the average number of months per city where droughts (or floods) occurred or did not occur from 2008 to 2020.

The *p* values were calculated by Kruskal-Wallis rank-sum tests for continuous variables and Pearson’s Chi-square test or Fisher’s exact test for categorical variables with p < 0.05 indicating statistical significance marked in bold.

HFRS: hemorrhagic fever with renal syndrome.

**Table 2:** Pooled exposure-response in RRs at specific percentiles between the meteorological factors and the monthly incidence rate of HFRS during different lag periods.

Index	Lag	Type I (95%CI)		Type II (95%CI)		Type III (95%CI)	
	(month)	RR (P25)	RR (P75)	RR (P25)	RR (P75)	RR (P25)	RR (P75)

Rainfall	**1**	1.039 (0.918, 1.176)	**0.871 (0.777, 0.976)**	1.019 (0.952, 1.090)	**0.906 (0.830, 0.989)**	1.013 (0.967, 1.060)	**0.921 (0.859, 0.988)**
	**2**	0.993 (0.904, 1.089)	0.912 (0.819, 1.016)	1.012 (0.944, 1.084)	0.979 (0.893, 1.074)	1.016 (0.956, 1.080)	0.932 (0.863, 1.007)
	**3**	**0.861 (0.763, 0.971)**	1.103 (0.975, 1.247)	1.017 (0.942, 1.099)	1.010 (0.915, 1.116)	0.978 (0.913, 1.047)	0.999 (0.915, 1.091)
		**SPI=−1**	**SPI=1**	**SPI=−1**	**SPI=1**	**SPI=−1**	**SPI=1**
SPI	**1**	**0.854 (0.791, 0.921)**	**1.114 (1.016, 1.222)**	0.972 (0.898, 1.051)	0.982 (0.922, 1.045)	**0.922 (0.865, 0.983)**	**1.133 (1.053, 1.220)**
	**2**	**0.803 (0.723, 0.893)**	**1.126 (1.022, 1.240)**	0.951 (0.857, 1.055)	0.978 (0.921, 1.037)	**0.866 (0.810, 0.927)**	**1.166 (1.063, 1.279)**
	**3**	**0.848 (0.758, 0.949)**	1.009 (0.900, 1.131)	0.945 (0.849, 1.051)	0.952 (0.883, 1.026)	**0.916 (0.843, 0.996)**	**1.113 (1.004, 1.235)**
		**SPI=−1.5**	**SPI=1.5**	**SPI=−1.5**	**SPI=1.5**	**SPI=−1.5**	**SPI=1.5**
SPI	**1**	**0.826 (0.747, 0.914)**	**1.158 (1.030, 1.303)**	0.973 (0.883, 1.073)	0.977 (0.907, 1.052)	**0.900 (0.836, 0.970)**	**1.241 (1.132, 1.360)**
	**2**	**0.688 (0.575, 0.823)**	**1.226 (1.073, 1.401)**	0.961 (0.835, 1.106)	0.977 (0.907, 1.052)	**0.850 (0.780, 0.926)**	**1.338 (1.184, 1.511)**
	**3**	**0.680 (0.544, 0.851)**	1.113 (0.935, 1.324)	0.962 (0.812, 1.140)	0.973 (0.888, 1.066)	0.896 (0.797, 1.007)	**1.296 (1.130, 1.487)**
Temperature	**1**	**1.358 (1.040, 1.774)**	**0.149 (0.113, 0.197)**	1.201 (0.990, 1.457)	**0.659 (0.547, 0.795)**	0.881 (0.675, 1.150)	**0.412 (0.331, 0.512)**
	**2**	0.752 (0.555, 1.021)	**0.232 (0.142, 0.379)**	**1.218 (1.081, 1.372)**	**0.651 (0.578, 0.734)**	**0.595 (0.483, 0.731)**	**0.560 (0.431, 0.728)**
	**3**	**0.392 (0.248, 0.618)**	**0.516 (0.339, 0.785)**	**1.253 (1.091, 1.438)**	**0.710 (0.656, 0.768)**	**0.358 (0.254, 0.506)**	**0.732 (0.567, 0.946)**
RH	**1**	**1.091 (1.003, 1.186)**	**0.841 (0.777, 0.910)**	1.023 (0.967, 1.082)	0.968 (0.903, 1.036)	**1.076 (1.037, 1.116)**	**0.858 (0.808, 0.912)**
	**2**	1.053 (0.990, 1.119)	**0.896 (0.817, 0.983)**	1.045 (0.977, 1.117)	**0.908 (0.850, 0.970)**	1.033 (0.993, 1.074)	0.963 (0.922, 1.006)
	**3**	**1.134 (1.061, 1.211)**	**0.872 (0.772, 0.984)**	1.034 (0.970, 1.101)	1.004 (0.937, 1.077)	**1.057 (1.010, 1.107)**	1.002 (0.955, 1.053)

The parameter estimations of three meteorological factors including monthly average temperature, monthly cumulative rainfall and monthly average RH were all transformed into percentile scale in analysis with the references at their respective medians, while the SPI retained its original scale with the reference at 0. The RR values of P25 and P75 were given for all the meteorological indicators, except that those of SPI were given at ±1 and ±1.5 to illustrate the lag effects of different wetness or dryness levels on HFRS incidence. The 95%CIs marked in bold indicated statistical significance.RR: relative risk. HFRS: hemorrhagic fever with renal syndrome. CI: confidence interval. RH: relative humidity. SPI: standardized precipitation index. P25: the 25th percentile. P75: the 75th percentile.

## Data Availability

Data will be made available on request.

## Highlights

1. Three endemic types of HFRS were determined by genotyping and hierarchical cluster.
2. The HTNV-dominant endemic area was related to higher incidence level of HFRS.
3. Persistent rain and flood affected HFRS incidence oppositely in HTNV-dominant area.
4. HFRS incidence in HTNV-dominant area was more vulnerable to climatic variation.
