# Supplementary material for: Dual seasonal pattern for hemorrhagic fever with renal syndrome and its potential determinants in China
Source: Sci Total Environ. Author manuscript; Available in PMC 2025 Nov 13. (PMC12614763; doi:10.1016/j.scitotenv.2022.160339)
Supplement: Supplementary appendix [file NIHMS2109443-supplement-Supplementary_appendix.docx]

**Supplementary appendix**

Dual seasonal pattern for hemorrhagic fever with renal syndrome and its potential determinants in China

**Table of Contents**

| **Page** | **Item** |
| --- | --- |
| 1−4 | **Supplementary Material and Methods** |
| 5 | **Supplementary References** |
| 6 | **Table S1.** Coding of the selected 76 cities in the current study |
| 7 | **Table S2.** Data collection and management involved in our study |
| 8 | **Table S3.** Description of land cover types |
| 9 | **Table S4.** Classification of extreme weather based on SPI |
| 10 | **Table S5.** Evaluation of urbanization level |
| 11−12 | **Table S6.** Basic characteristics of the selected 76 cities in the mainland of China, 2008−2020 |
| 13 | **Table S7.** Environmental characteristics of three types of HFRS endemic cities in the mainland of China, 2008−2020 |
| 14 | **Fig. S1.** Temporal pattern of HFRS incidence in three types of endemic cities stratified by age and gender |
| 15 | **Fig. S2.** Temporal pattern of HFRS in 76 selected cities of three endemic types by month and by year |
| 16 | **Table S8.** Parameter estimation for pooled exposure-response curves between the habitat factors and the annualized incidence rates of HFRS based on GAMM |
| 17 | **Fig. S3.** Pooled exposure-response curves between the other habitat factors and the annualized incidence rates of HFRS for types I or II based on GAMM |
| 18−19 | **Table S9.** Associations of habitat factors with the changing trends of annualized incidence rates of HFRS for each types I-III city |
| 20−25 | **Table S10.** Monthly moving average of meteorological factors of each types I-III city during different lag periods |
| 26 | **Fig. S4.** Pooled exposure-response in relative risks between the meteorological factors and the monthly incidence rates of HFRS during different lag periods in Type I cities |
| 27 | **Fig. S5.** Pooled exposure-response in relative risks between the meteorological factors and the monthly incidence rates of HFRS during different lag periods in Type II cities |
| 28 | **Fig. S6.** Pooled exposure-response in relative risks between the meteorological factors and the monthly incidence rates of HFRS during different lag periods in Type III cities |

**Supplementary Material and Methods**

***Literature review and data extraction***

A comprehensive database of hantavirus was mainly collected from literature review and GenBank database. We searched PubMed, Web of Science, China National Knowledge Infrastructure, WanFang database, and VIP databases with the terms “rodent OR rodent associated OR rodent borne”, “Hantavirus” and “China”. Cross-check was carefully performed for data to ensure the compliance with extraction criteria. Each article was strictly reviewed by two researchers independently to extract the following information with a standard sheet: study date, study location, rodent species identified, hantavirus species, laboratory methods, and detection results for hantavirus. The hantavirus was detected with antibody-based serological tests (e.g. ELISA, IFA), isolation, or detection of RNA (e.g. RT-PCR). The corresponding authors were contacted for clarification when articles lacking key information. Only studies with clearly identifiable results, i.e., presence or absence, time and location of rodent species or hantavirus were included in our database. If more than one species of hantavirus were found in the same study, a record was created for each pathogen in our database. The data from all sources were integrated to form one database at the county level for the final analyses.

***The estimation of Standardized Precipitation Index (SPI)***

Since the rainfall is generally a biased rather than a normal distribution, the index uses Gamma distribution to describe the periodic variation of rainfall. After fitting the historic records of rainfall to the Gamma function, the probability of rainfall data point is obtained and used along with the inverse normal to calculate the deviation for a standardized normal distributed probability density (Mckee et al., 1993). This value is the SPI which has been widely applied to drought and flood surveillance by the National Climate Center of China Meteorological Administration (China Meteorological Administration, 2017). The SPI can be calculated using the following formula (China Meteorological Administration, 2017):

$$SPI=S(t-\frac{2.52+0.8t+0.01t^{2}}{1+1.43t+0.19t^{2}+0.001t^{3}})$$

where $t=\sqrt{ln(1/F^{2})}$, *F* is the probability distribution obtained based on Gamma probability density function. When *F* >0.5, *F* = 1−*F*, *S* = 1, and when *F* ≤0.5, *S* = −1.

***Equations of entropy weights calculation***

Firstly, the original value *x_i,j_* of the *j*-th indicator in the *i*-th year (*i* = 1, 2, ..., 13; *j* = 1, 2, 3) was normalized to *X_i,j_* using Equation (1), where 0.0001 represents an additional minimum value to ensure the result greater than 0 so that the subsequent logarithmic transformation can be performed. Then the proportion of *X_i,j_* was calculated by Equation (2), and the entropy of *j*-th indicator was determined with Equation (3). Finally, the entropy weight coefficient of each indicator could be obtained from Equation (4).

$X_{i,j}=\frac{x_{i,j}-x_{j\min}}{x_{j\max}-x_{j\min}}\text{+}0.0001$ (1)

$P_{i,j}=\frac{X_{i,j}}{\sum_{i=1}^{13} X_{i,j}}$ (2)

$e_{j}=-\frac{1}{\ln m}\sum_{i=1}^{13} P_{i,j}\ln P_{i,j}$ (3)

$\omega_{j}=\frac{1-e_{j}}{\sum_{j=1}^{3} (1-e_{j})}$ (4)

Here *x_j max_* and *x_j min_* is the maximum and minimum value of the *j*-th indicator in all years respectively.

***Generalized additive mixed model (GAMM)***

The GAMM framework was used to model habitat properties associated with different seasonal patterns of HFRS, with random effect by cities added to adjust for the impact due to sampling from different cities. The GAMM can be written as follows:

$$\log[E(Y_{t,k})]=\alpha+b_{k}+\sum_{i=1} s_{i}(X_{i,t,k})+s(year)+\varepsilon_{t,k}$$

In the model, *Y_t,k_* is used to represent the number of HFRS cases on year *t* in city *k* of each type with *α* as the intercept, *b_k_* as the random effect of city *k*, *s(year)* as adjustment for time trend and *ε_t,k_* as the residuals of model. *X_i,t,k_* refer to the annual habitat indicators in city *k* with *s_i_* as the additive smooth functions accordingly. Correlation analysis was used to identify whether there was collinearity among the explanatory variables. If a relatively high correlation between any two variables is observed (the correlation coefficient is more than 0.7), the one with larger AIC is then dropped.

***Distributed lag nonlinear model (DLNM) analysis***

The DLNM framework allows the estimation of the nonlinear association between environmental exposure at each unit time interval (day/week/month) and HFRS, while adjusting for exposures during the other time intervals (Gasparrini, 2014; Gasparrini, 2011). The DLNM can be written as follows (Gasparrini et al., 2012):

$$\log[E(Y_{t,k})]=\alpha_{k}+s(Var_{t,l,k};\theta_{k})+ns(Time,df)+\sum_{i=1}^{3} \beta_{i,l,k}X_{i,l,k}$$

where *Y_t,k_* represents the number of HFRS cases on month *t* in city *k* of each type. *α_k_* is the intercept of city *k*. The exposure *Var_t,l,k_* corresponds to one of the four monthly indicators over lag 0−*l* (1, 2 and 3 months were selected as the lag periods *l* respectively), computed as the moving average on month t and on the previous *l* months, in order to account for lag effects. The association between *Y_t,k_* and *Var_t,l,k_* is defined by the vector ***θ_k_*** and specified using a natural cubic spline as the exposure-response function *s*. The *p-*length vector of estimated parameters ${\hat{\boldsymbol{\theta}}}_{\boldsymbol{k}}$ and accompany *p* × *p* estimated (co)variance matrix ***S_k_*** for each city, applied in the subsequent analysis, are the best linear unbiased prediction derived from the true unknown association ***θ_k_*** (Verbeke, 1997). Each month of the study period, represented by variable *Time*, is used in the model to adjust for the long-term and seasonal trends with a natural cubic spline (*ns*) as well. *X_i,l,k_* refer to the other three (*i* = 1, 2, 3) monthly indicators in city *k* respectively, which are calculated as the moving average from month *t−l* to month *t* to control for their confounding effects with *β_i,l,k_* as the coefficients. The optimal degrees of freedom (*df*) for the spline functions are selected based on minimizing Quasi Akaike Information Criterion (QAIC) (Gasparrini, 2014).

***Multivariate meta-analysis***

Meta-analytic techniques have been applied more widely as a research (each city can be regarded as a research in our study) synthesis approach, with the aim of summarizing an average association in a single parameter estimate across studies and to explore the degree and sources of heterogeneity (Sutton, 2008). However, the association estimated from DLNM, which are usually represented with functions defined by multiple parameters, requires more complicated meta-analytic methods to deal with the multivariate nature of the summary calculation. Multivariate meta-analysis, an approach initially developed to integrate multiple related outcomes in randomized controlled trials (Arends et al., 2003), provides a way to construct the model.

A model for random-effect multivariate meta-analysis can be written as follows (Gasparrini et al., 2012):

$$\widehat{\theta}_{k}\sim N_{p}(\theta,S_{k}+\Psi)$$

The variation contained in the above model consists of two parts: within-city and between-city. In the within-city component, the estimated ${\hat{\boldsymbol{\theta}}}_{\boldsymbol{k}}$ is assumed to be sampled from a multivariate normal distribution ***N_p_***(***θ_k_***, ***S_k_***). In the between-city component, ***θ_k_*** is assumed sampled from ***N_p_***(***θ***, ***Ψ***), where ***Ψ*** represents the unknown between-city (co)variance matrix and ***θ*** represents the average exposure-response association defined in DLNM, namely the average city-specific estimates for each type.

**Supplementary References**

Arends L. R., Voko Z., Stijnen T., 2003. Combining multiple outcome measures in a meta-analysis: an application. Stat Med 22(8): 1335-1353. https://doi.org/10.1002/sim.1370.

China Meteorological Administration, 2017. Grades of meteorological drought. http://c.gb688.cn/bzgk/gb/showGb?type=online&hcno=D2281945A96E8185F67EDC9E7A698049/ (accessed 15 November 2022).

Gasparrini A., 2011. Distributed Lag Linear and Non-Linear Models in R: The Package dlnm. J. Stat. Softw. 43(8): 1-20.

Gasparrini A., 2014. Modeling exposure-lag-response associations with distributed lag non-linear models. Stat. Med. 33(5): 881-899. https://doi.org/10.1002/sim.5963.

Gasparrini A., Armstrong B., Kenward M. G., 2012. Multivariate meta-analysis for non-linear and other multi-parameter associations. Stat. Med. 31(29): 3821-3839. https://doi.org/10.1002/sim.5471.

Mckee T. B., Doesken N. J., Kleist J., 1993. The Relationship of Drought Frequency and Duration to Time Scales.

Sutton A. J., Higgins J. P., 2008. Recent developments in meta-analysis. Stat. Med. 27(5): 625-650. https://doi.org/10.1002/sim.2934.

Verbeke G., 1997. Linear mixed models for longitudinal data. Linear mixed models in practice: Springer; 63-153.

**Table S1. Coding of the selected 76 cities in the current study.**

| **Province (autonomous region)** | **City (code)** |
| --- | --- |
| Heilongjiang | Harbin (2301), Qiqihar (2302), Jixi (2303), Hegang (2304), Shuangyashan (2305), Daqing (2306), Yichun (2307), Jiamusi (2308), Mudanjiang (2310), Heihe (2311), Suihua (2312), |
| Jilin | Changchun (2201), Jilin (2202), Siping (2203), Tonghua (2205), Baishan (2206), Baicheng (2208), Yanbian (2224) |
| Liaoning | Shenyang (2101), Fushun (2104), Benxi (2105), Dandong (2106), Jinzhou (2107), Yingkou (2108), Tieling (2112), Chaoyang (2113), Huludao (2114) |
| Inner Mongolia | Hulunbuir (1507) |
| Hebei | Tangshan (1302), Qinhuangdao (1303) |
| Shandong | Jinan (3701), Qingdao (3702), Zibo (3703), Yantai (3706), Weifang (3707), Jining (3708), Rizhao (3711), Linyi (3713) |
| Shaanxi | Xi’an (6101), Baoji (6103), Xianyang (6104), Weinan (6105) |
| Henan | Zhumadian (4117) |
| Jiangsu | Lianyungang (3207), Yancheng (3209) |
| Zhejiang | Ningbo (3302), Shaoxing (3306), Quzhou (3308), Taizhou (3310), Lishui (3311) |
| Jiangxi | Nanchang (3601), Yichun (3609), Fuzhou (3610), Shangrao (3611) |
| Fujian | Fuzhou (3501), Quanzhou (3505), Nanping (3507), Ningde (3509) |
| Hubei | Xiangyang (4206), Jingmen (4208), Jingzhou (4210) |
| Hunan | Changsha (4301), Xiangtan (4303), Hengyang (4304), Shaoyang (4305), Yiyang (4309), Chenzhou (4310), Huaihua (4312), Loudi (4313) |
| Sichuan | Liangshan (5134) |
| Guizhou | Zunyi (5203) |
| Guangdong | Guangzhou (4401), Shenzhen (4403), Foshan (4406) |
| Yunnan | Chuxiong (5323), Dali (5329) |

**Table S2. Data collection and management involved in our study.**

| **Data item** | **Indicator** | **Note** | **Data period** | **Data source** |
| --- | --- | --- | --- | --- |
| Demographic data | Population, population density, proportion of urban population | Annual data at the city level. | 2008‒2020 | National and Local Bureau of Statistics |
| Socio-economic data | GDP | Annual data at the city level. | 2008‒2020 | National and Local Bureau of Statistics |
| Elevation data | Elevation | Raster-type map lay with a spatial resolution of 1 km. | 2010 | Resource and Environment Science and Data Center (https://www.resdc.cn/) |
| NDVI data | NDVI | Annual raster-type map lay with a spatial resolution of 1 km. | 2008‒2020 | Resource and Environment Science and Data Center (https://www.resdc.cn/) |
| Land cover data | Rainfed cropland, irrigated cropland, broad-leaved tree, needle-leaved tree, shrubland, grassland, wetland, built-up land | Annual raster-type map lay with a spatial resolution of 0.3 km. | 2008‒2020 | European Space Agency (https://www.esa.int) |
| Meteorological data | Temperature, rainfall, RH | Annual raster-type map lay with a spatial resolution of 5 km. | 2008‒2020 | 613 weather surveillance stations (http://data.cma.cn/) |

GDP: gross domestic product. NDVI: normalized difference vegetation index. RH: relative humidity.

**Table S3. Description of land cover types.**

| **Land cover** | **Description** |
| --- | --- |
| Rainfed cropland | Rainfed shrub crops/rainfed tree crops/rainfed herbaceous crops |
| Irrigated cropland | Irrigated tree crops/irrigated shrub crops/irrigated herbaceous crops/post-flooding cultivation of herbaceous crops |
| Broad-leaved tree | Broad-leaved evergreen closed to open trees/broad-leaved semi-deciduous closed to open trees/broad-leaved deciduous closed to open trees |
| Needle-leaved tree | Needle-leaved evergreen closed to open trees/needle-leaved deciduous closed to open trees |
| Shrubland | Broad-leaved closed to open shrubland (thicket) |
| Grassland | Herbaceous closed to very open vegetation |
| Wetland | Closed to open (100−40%) broad-leaved trees on temporarily or permanently flooded land, water quality: fresh water/closed to open (100−40%) broad-leaved trees on permanently flooded land (with daily variations), water quality: saline water or brackish water/closed to open (100−40%) semi-deciduous shrubland on permanently flooded land (with daily variations), water quality: saline water or brackish water/closed to open shrubs on temporarily or permanently flooded land/closed to open herbaceous vegetation on permanently flooded land/closed to open shrubs on waterlogged soil/closed to open herbaceous vegetation on waterlogged soil water quality: fresh, brackish or saline water |
| Built-up land | Artificial surfaces and neighboring areas |

**Table S4. Classification of extreme weather based on SPI.** An extreme weather with SPI less than −1.5 is defined as a drought, and with SPI more than 1.5 is defined as a flood.

| **Types** | **SPI** |
| --- | --- |
| Extreme wetness | ≥2.0 |
| Severe wetness | 1.5 to 1.99 |
| Moderate wetness | 1.0 to 1.49 |
| Normal | −0.99 to 0.99 |
| Moderate dryness | −1.49 to −1.0 |
| Severe dryness | −1.99 to −1.5 |
| Extreme dryness | ≤−2.0 |

SPI: standardized precipitation index.

**Table S5. Evaluation of urbanization level.**

| **First-grade indicator** |  | **Second-grade indicator** |  | **Weight** |
| --- | --- | --- | --- | --- |
| Urbanization of population |  | Proportion of urban population (%) |  | 0.216 |
| Urbanization of land use |  | Areas of built-up land per capita (m^2^) |  | 0.348 |
| Economic urbanization |  | GDP per capita (Chinese yuan) |  | 0.436 |

GDP: gross domestic product.

**Table S6. Basic characteristics of the selected 76 cities in the mainland of China, 2008−2020.**

| City | Province | Type | No. of cases | Incidence rate (per 100,000 people) | No. of deaths | Case fatality rate (%) | Trend |
| --- | --- | --- | --- | --- | --- | --- | --- |
| Qiqihar^†^ | Heilongjiang | I | 2,137 | 2.986 | 25 | 1.170 | Increase |
| Jixi^†^ | Heilongjiang | I | 1,530 | 6.172 | 15 | 0.980 | Decrease |
| Yichun | Heilongjiang | I | 598 | 3.773 | 8 | 1.338 | Fluctuation |
| Heihe^†^ | Heilongjiang | I | 1,585 | 7.074 | 22 | 1.388 | Decrease |
| Fushun | Liaoning | I | 1,497 | 5.334 | 4 | 0.267 | Fluctuation |
| Hulunbuir^†^ | Inner Mongolia | I | 1,103 | 3.051 | 12 | 1.088 | Fluctuation |
| Yantai | Shandong | I | 1,228 | 1.347 | 62 | 5.049 | Fluctuation |
| Weifang | Shandong | I | 2,956 | 2.444 | 16 | 0.541 | Fluctuation |
| Rizhao | Shandong | I | 1,620 | 4.557 | 11 | 0.679 | Decrease |
| Linyi | Shandong | I | 1,833 | 1.382 | 8 | 0.436 | Decrease |
| Zhumadian | Henan | I | 649 | 0.694 | 11 | 1.695 | Fluctuation |
| Xi’an^†^ | Shaanxi | I | 11,333 | 10.228 | 78 | 0.688 | Fluctuation |
| Baoji^†^ | Shaanxi | I | 3,220 | 6.437 | 13 | 0.404 | Fluctuation |
| Xianyang | Shaanxi | I | 4,106 | 6.040 | 11 | 0.268 | Fluctuation |
| Weinan | Shaanxi | I | 3,562 | 5.053 | 7 | 0.197 | Fluctuation |
| Lianyungang | Jiangsu | I | 1,057 | 1.831 | 31 | 2.933 | Decrease |
| Yancheng | Jiangsu | I | 728 | 0.756 | 18 | 2.473 | Increase |
| Taizhou^†^ | Zhejiang | I | 1,192 | 1.554 | 1 | 0.084 | Decrease |
| Daqing | Heilongjiang | II | 519 | 1.340 | 2 | 0.385 | Fluctuation |
| Changchun^†^ | Jilin | II | 1,322 | 1.316 | 3 | 0.227 | Decrease |
| Jilin^†^ | Jilin | II | 932 | 1.611 | 4 | 0.429 | Decrease |
| Siping | Jilin | II | 658 | 1.438 | 0 | 0 | Decrease |
| Baicheng | Jilin | II | 921 | 3.408 | 2 | 0.217 | Increase |
| Jinzhou^†^ | Liaoning | II | 1,730 | 4.396 | 5 | 0.289 | Fluctuation |
| Yingkou | Liaoning | II | 667 | 2.125 | 0 | 0 | Fluctuation |
| Chaoyang | Liaoning | II | 609 | 1.381 | 5 | 0.821 | Increase |
| Huludao | Liaoning | II | 2,884 | 7.942 | 6 | 0.208 | Fluctuation |
| Tangshan^†^ | Hebei | II | 2,621 | 2.614 | 1 | 0.038 | Fluctuation |
| Qinhuangdao^†^ | Hebei | II | 3,019 | 7.820 | 4 | 0.132 | Fluctuation |
| Jining | Shandong | II | 1,107 | 1.005 | 4 | 0.361 | Fluctuation |
| Fuzhou^†^ | Fujian | II | 690 | 0.694 | 0 | 0 | Fluctuation |
| Quanzhou^†^ | Fujian | II | 764 | 0.694 | 3 | 0.393 | Increase |
| Nanping^†^ | Fujian | II | 1,341 | 3.262 | 2 | 0.149 | Increase |
| Ningde^†^ | Fujian | II | 554 | 1.433 | 0 | 0 | Fluctuation |
| Shaoyang | Hunan | II | 916 | 0.975 | 2 | 0.218 | Decrease |
| Liangshan | Sichuan | II | 865 | 1.474 | 2 | 0.231 | Increase |
| Guangzhou^†^ | Guangdong | II | 1,519 | 0.767 | 4 | 0.263 | Fluctuation |
| Shenzhen | Guangdong | II | 548 | 0.314 | 4 | 0.730 | Fluctuation |
| Foshan | Guangdong | II | 668 | 0.713 | 0 | 0 | Fluctuation |
| Chuxiong | Yunnan | II | 679 | 1.897 | 2 | 0.295 | Increase |
| Dali | Yunnan | II | 1,068 | 2.313 | 4 | 0.375 | Increase |
| Harbin | Heilongjiang | III | 2,620 | 2.067 | 18 | 0.687 | Decrease |
| Hegang | Heilongjiang | III | 1,070 | 7.647 | 6 | 0.561 | Fluctuation |
| Shuangyashan | Heilongjiang | III | 2,067 | 10.555 | 18 | 0.871 | Decrease |
| Jiamusi | Heilongjiang | III | 2,993 | 8.832 | 14 | 0.468 | Decrease |
| Mudanjiang^†^ | Heilongjiang | III | 2,163 | 5.772 | 12 | 0.555 | Decrease |
| Suihua | Heilongjiang | III | 1,592 | 2.224 | 3 | 0.188 | Fluctuation |
| Tonghua | Jilin | III | 1,372 | 4.456 | 3 | 0.219 | Decrease |
| Baishan^†^ | Jilin | III | 947 | 5.536 | 14 | 1.478 | Decrease |
| Yanbian^†^ | Jilin | III | 1,442 | 4.914 | 12 | 0.832 | Decrease |
| Shenyang^†^ | Liaoning | III | 963 | 0.896 | 4 | 0.415 | Decrease |
| Benxi | Liaoning | III | 699 | 3.148 | 11 | 1.574 | Fluctuation |
| Dandong^†^ | Liaoning | III | 884 | 2.853 | 2 | 0.226 | Fluctuation |
| Tieling | Liaoning | III | 1,096 | 2.816 | 4 | 0.365 | Fluctuation |
| Jinan | Shandong | III | 679 | 0.752 | 10 | 1.473 | Fluctuation |
| Qingdao^†^ | Shandong | III | 2,300 | 1.908 | 60 | 2.609 | Decrease |
| Zibo^†^ | Shandong | III | 965 | 1.665 | 10 | 1.036 | Fluctuation |
| Ningbo | Zhejiang | III | 1,314 | 1.723 | 1 | 0.076 | Decrease |
| Shaoxing | Zhejiang | III | 700 | 1.216 | 3 | 0.429 | Decrease |
| Quzhou | Zhejiang | III | 576 | 2.046 | 1 | 0.174 | Fluctuation |
| Lishui | Zhejiang | III | 618 | 2.079 | 3 | 1.151 | Decrease |
| Nanchang | Jiangxi | III | 782 | 1.173 | 9 | 1.252 | Fluctuation |
| Yichun | Jiangxi | III | 3,474 | 4.860 | 53 | 0.124 | Fluctuation |
| Fuzhou | Jiangxi | III | 559 | 1.077 | 7 | 2.117 | Increase |
| Shangrao | Jiangxi | III | 1,615 | 1.868 | 2 | 2.143 | Fluctuation |
| Xiangyang | Hubei | III | 803 | 1.095 | 17 | 1.832 | Fluctuation |
| Jingmen | Hubei | III | 560 | 1.473 | 12 | 0.901 | Fluctuation |
| Jingzhou^†^ | Hubei | III | 1,201 | 1.567 | 22 | 0.291 | Increase |
| Changsha | Hunan | III | 1,553 | 1.674 | 14 | 0.957 | Increase |
| Xiangtan | Hunan | III | 687 | 1.885 | 2 | 0.896 | Fluctuation |
| Hengyang | Hunan | III | 627 | 0.661 | 6 | 0.123 | Fluctuation |
| Yiyang | Hunan | III | 558 | 0.971 | 5 | 0 | Increase |
| Chenzhou | Hunan | III | 813 | 1.330 | 1 | 1.226 | Fluctuation |
| Huaihua | Hunan | III | 504 | 0.806 | 0 | 0.756 | Fluctuation |
| Loudi | Hunan | III | 1,060 | 2.118 | 13 | 1.170 | Fluctuation |
| Zunyi | Guizhou | III | 661 | 0.809 | 5 | 0.980 | Decrease |

† represents that the city was included in the analysis of hantavirus genospecies.

**Table S7. Environmental characteristics of three types of HFRS endemic cities in the mainland of China, 2008−2020.**

|  | **Type I cities (N=18)** |  | **Type II cities (N=23)** |  | **Type III cities (N=35)** |  | **p value** |
| --- | --- | --- | --- | --- | --- | --- | --- |
| Elevation (m), median (IQR) | 225.030 (112.067, 615.508) |  | 222.808 (127.207, 481.683) |  | 214.291 (136.489, 395.615) |  | 0.937 |
| Population density (per km^2^), median (IQR) | 501.471 (109.604, 579.930) |  | 289.318 (140.239, 695.523) |  | 256.231 (151.014,581.350) |  | 0.123 |
| NDVI, median (IQR) | 0.774 (0.730, 0.828) |  | 0.768 (0.716, 0.808) |  | 0.805 (0.767, 0.858) |  | <0.001 |
| Areas of rainfed cropland per capita (m^2^), median (IQR) | 335.966 (140.627, 641.352) |  | 257.561 (50.422, 579.702) |  | 175.604 (89.054, 361.946) |  | <0.001 |
| Areas of irrigated cropland per capita (m^2^), median (IQR) | 359.260 (224.433, 565.356) |  | 185.172 (92.172, 387.067) |  | 341.423 (122.324, 567.880) |  | <0.001 |
| Areas of broad-leaved tree per capita (m^2^), median (IQR) | 73.386 (2.603, 3,442.169) |  | 69.188 (23.379, 619.114) |  | 541.718 (167.121, 2,523.758) |  | <0.001 |
| Areas of needle-leaved tree per capita (m^2^), median (IQR) | 18.480 (4.678, 288.079) |  | 28.271 (8.317, 428.616) |  | 62.495 (17.379, 429.331) |  | <0.001 |
| Areas of shrubland per capita (m^2^), median (IQR) | 0.017 (0.000, 0.686) |  | 0.516 (0.000, 2.461) |  | 0.933 (0.087, 2.471) |  | <0.001 |
| Areas of grassland per capita (m^2^), median (IQR) | 34.414 (16.125, 189.357) |  | 40.512 (2.650, 403.008) |  | 6.865 (2.178, 26.411) |  | <0.001 |
| Areas of wetland per capita (m^2^), median (IQR) | 7.164 (0.651, 32.915) |  | 4.370 (1.937, 19.769) |  | 5.269 (1.985, 46.612) |  | 0.302 |
| Areas of built-up land per capita (m^2^), median (IQR) | 117.868 (77.107, 191.681) |  | 106.895 (52.055, 135.214) |  | 72.722 (42.099, 135.170) |  | <0.001 |

The p values were calculated by Kruskal-Wallis rank-sum tests.

HFRS: hemorrhagic fever with renal syndrome. IQR: interquartile range. NDVI: normalized difference vegetation index.

**Fig. S1. Temporal pattern of HFRS incidence in three types of endemic cities stratified by age and gender.** The peaking time among subgroups are marked with red arrows in types I and II. HFRS: hemorrhagic fever with renal syndrome.

**
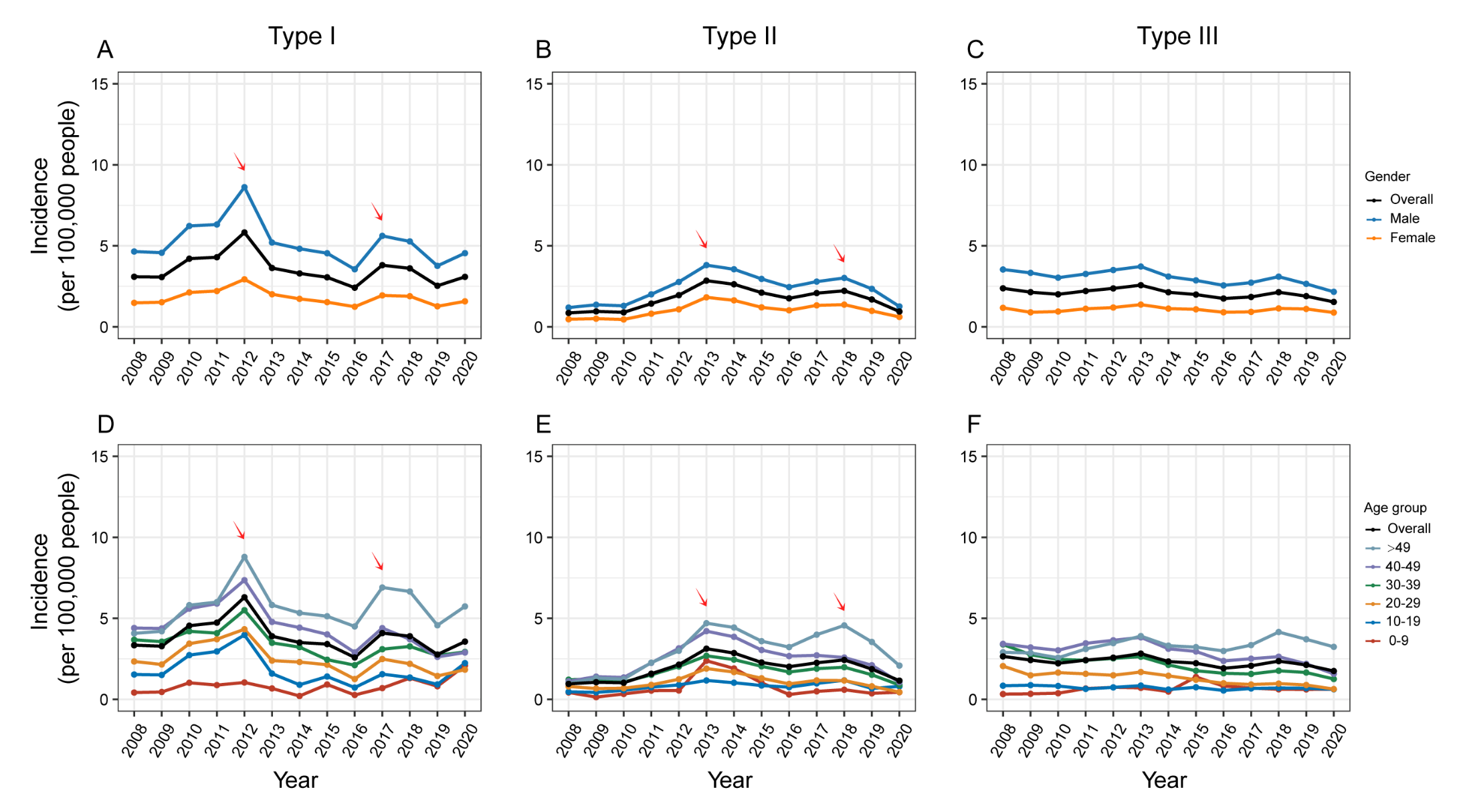
**

**Fig. S2.** **Temporal pattern of HFRS in 76 selected cities of three endemic types by month and by year.** (A) The average monthly proportion of HFRS cases. (B) Annual incidence of HFRS cases from 2008 to 2020. HFRS: hemorrhagic fever with renal syndrome.

**
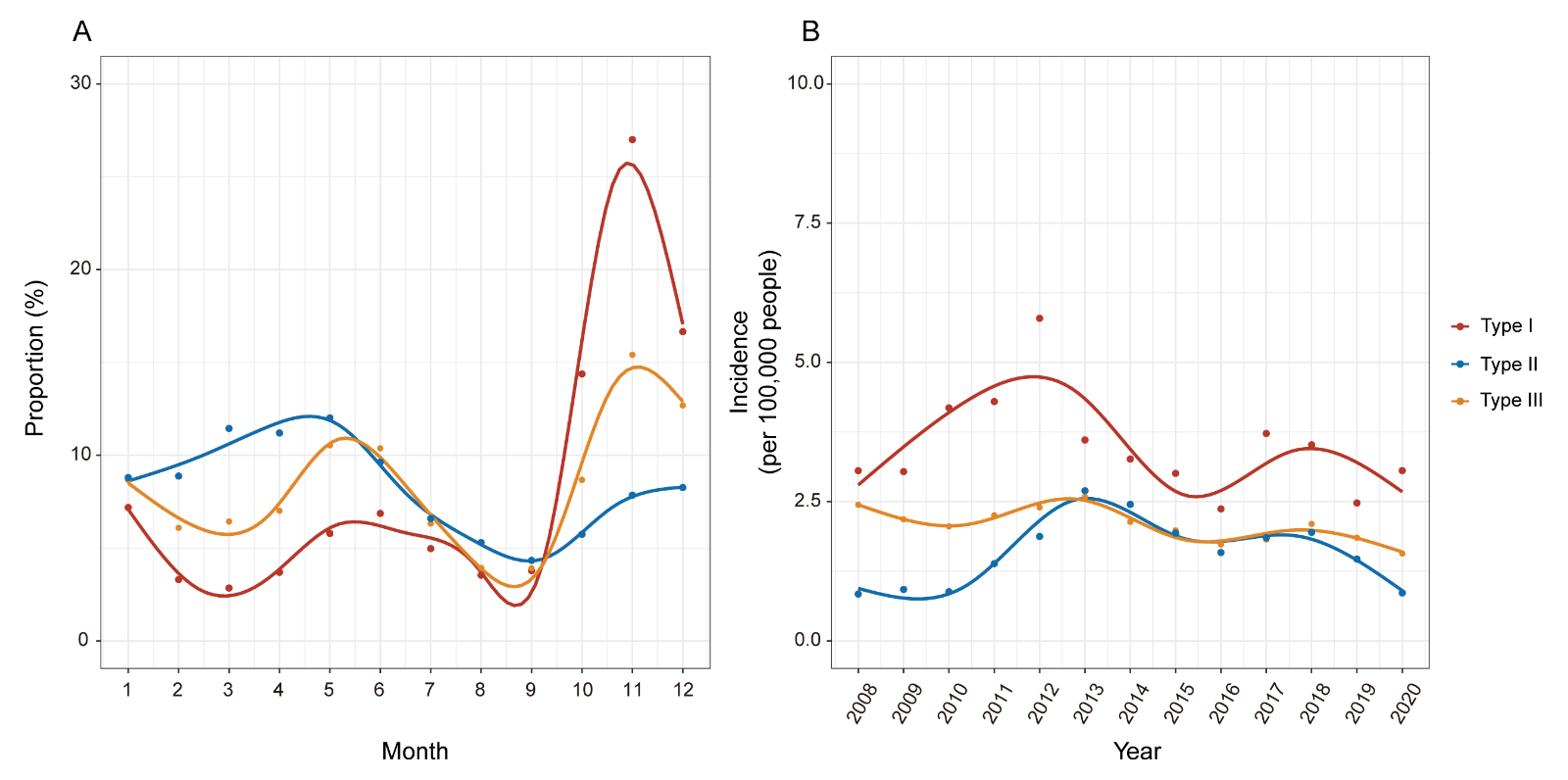
**

**Table S8. Parameter estimation for pooled exposure-response curves between the habitat factors and the annualized incidence rates of HFRS based on GAMM.** Broad-leaved tree, shrubland and grassland were excluded because of their collinearity with NDVI, needle-leaved tree and wetland for Type I, respectively, while broad-leaved tree and elevation were excluded because of their collinearity with shrubland and needle-leaved tree for Type II, respectively.

| **Smooth terms** | **Type I cities** | | |  | **Type II cities** | | |
| --- | --- | --- | --- | --- | --- | --- | --- |
|  | **EDF** | **F value** | **p value** |  | **EDF** | **F value** | **p value** |
| s(Rainfed cropland) | 1.995 | 90.189 | <0.001 |  | 1.979 | 204.135 | <0.001 |
| s(Irrigated cropland) | 2.989 | 179.096 | <0.001 |  | 1.974 | 237.333 | <0.001 |
| s(Broad-leaved tree) | - | - | - |  | - | - | - |
| s(Needle-leaved tree) | 2.995 | 414.526 | <0.001 |  | 1.987 | 46.582 | <0.001 |
| s(Shrubland) | - | - | - |  | 1.993 | 98.789 | <0.001 |
| s(Grassland) | - | - | - |  | 1.999 | 443.179 | <0.001 |
| s(wetland) | 2.590 | 380.066 | <0.001 |  | 1.984 | 34.402 | <0.001 |
| s(Elevation) | 1.652 | 1753.169 | <0.001 |  | - | - | - |
| s(NDVI) | 2.945 | 97.847 | <0.001 |  | 1.000 | 425.363 | <0.001 |
| s(Urbanization index) | 2.946 | 238.079 | <0.001 |  | 3.840 | 50.018 | <0.001 |

HFRS: hemorrhagic fever with renal syndrome. EDF: effective degrees of freedom of the smooth function term (EDF >1 indicates nonlinear association). NDVI: normalized difference vegetation index.

**Fig. S3. Pooled exposure-response curves between the other habitat factors and the annualized incidence rates of HFRS for types I or II based on GAMM.** (A) Elevation. (B) Shrubland. (C) Grassland. The x-axis indicates the observed values of the habitat factors, while the y-axis indicates the contribution of the smooth term to the fitted values with the EDF in parentheses. The RRs were marked above the curves with their 95%CIs in parentheses, which were calculated based on the piecewise linear Poisson regression.

HFRS: hemorrhagic fever with renal syndrome. GAMM: generalized additive mixed model. RR: relative risk. CI: confidence interval. EDF: effective degrees of freedom of the smooth function term (EDF >1 indicates nonlinear association).

**
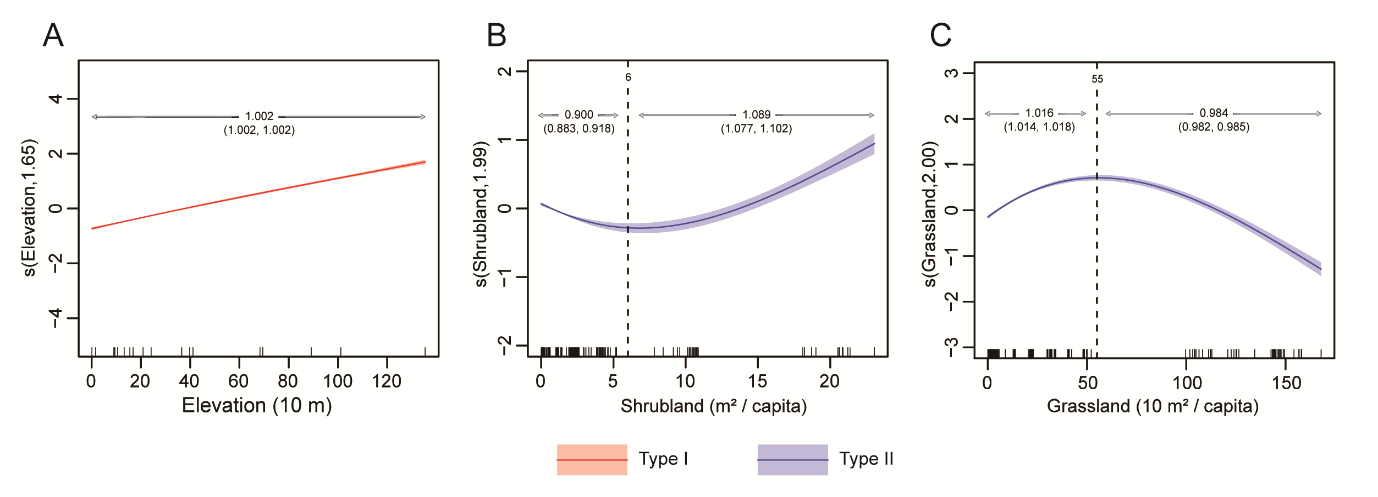
**

**Table S9. Associations of habitat factors with the changing trends of annualized incidence rates of HFRS for each types I-III city.**

|  | **Type I cities** | | | |  | **Type II cities** | | | |
| --- | --- | --- | --- | --- | --- | --- | --- | --- | --- |
|  | **Increase** | **Fluctuation** | **Decrease** | **p value** |  | **Increase** | **Fluctuation** | **Decrease** | **p value** |
| Elevation (m),  median (IQR) | 105.128  (53.241, 157.016) | 547.407  (178.580, 842.280) | 161.819  (137.763, 222.941) | 0.128 |  | 501.793  (454.188, 2,082.521) | 127.207  (82.669, 226.617) | 297.381  (201.631, 435.982) | 0.008 |
| NDVI,  median (IQR) | 0.802  (0.774, 0.835) | 0.771  (0.716, 0.833) | 0.770  (0.745, 0.821) | 0.054 |  | 0.759  (0.730, 0.808) | 0.755  (0.680, 0.781) | 0.844  (0.825, 0.858) | p<0.001 |
| Areas of rainfed cropland per capita (m^2^), median (IQR) | 1,019.260  (494.100, 1,562.335) | 260.909  (216.280, 438.799) | 388.725  (236.303, 600.297) | p<0.001 |  | 185.172  (91.363, 198.200 | 109.970  (63.407, 315.105) | 418.261  (358.852, 493.769) | p<0.001 |
| Areas of irrigated cropland per capita (m^2^),  median (IQR) | 182.852  (129.646, 234.735) | 607.279  (225.007, 870.465) | 253.623  (140.627, 622.305) | p<0.001 |  | 593.575  (260.252, 715.056) | 167.406  (46.177, 410.565) | 39.166  (21.139, 107.581) | p<0.001 |
| Areas of broad-leaved tree per capita (m^2^),  median (IQR) | 45.420  (0.000, 93.415) | 117.931  (7.897, 3,979.295) | 75.893  (1.155, 3,442.169) | p<0.001 |  | 623.285  (18.730, 1,195.357) | 26.321  (13.094, 124.243) | 299.487  (115.772, 1,225.846) | p<0.001 |
| Areas of needle-leaved tree per capita (m^2^),  median (IQR) | 2.273  (0.000, 5.676) | 23.106  (5.803, 288.079) | 67.291  (4.713, 456.476) | p<0.001 |  | 2,108.391  (31.936, 3,653.217) | 17.868  (8.448, 48.179) | 9.362  (2.446, 120.181) | p<0.001 |
| Areas of shrubland per capita (m^2^),  median (IQR) | 0.000  (0.000, 0.000) | 0.187  (0.000, 2.765) | 0.090  (0.000, 0.534) | p<0.001 |  | 1.283  (1.072, 4.468) | 0.050  (0.000, 2.041) | 1.321  (0.127, 4.470) | p<0.001 |
| Areas of grassland per capita (m^2^),  median (IQR) | 156.519  (21.623, 296.038) | 31.079  (5.171, 65.690) | 36.153  (27.034, 189.357) | 0.013 |  | 336.129  (2.650, 1,441.300) | 24.334  (5.178, 272.672) | 30.072  (18.962, 38.344) | p<0.001 |
| Areas of wetland per capita (m^2^),  median (IQR) | 167.934  (10.212, 335.437) | 5.307  (0.651, 19.792) | 5.594  (0.442, 37.443) | p<0.001 |  | 4.053  (2.112, 5.294) | 11.283  (1.878, 23.733) | 5.955  (1.863, 10.360) | 0.004 |
| Areas of built-up land per capita (m^2^),  median (IQR) | 169.042  (67.808, 265.778) | 105.329  (70.832, 160.061) | 133.211  (105.110, 219.440) | p<0.001 |  | 53.098  (29.652, 84.116) | 123.230  (81.823, 175.960) | 113.486  (60.706, 129.372) | p<0.001 |

HFRS: hemorrhagic fever with renal syndrome. IQR: interquartile range.

**Table S9 (Continued).**

|  | **Type III cities** | | | |
| --- | --- | --- | --- | --- |
|  | **Increase** | **Fluctuation** | **Decrease** | **p value** |
| Elevation (m), median (IQR) | 189.458 (142.892, 210.385) | 215.009 (171.520, 339.834) | 257.605 (131.035, 625.779) | 0.449 |
| NDVI, median (IQR) | 0.781 (0.755, 0.790) | 0.799 (0.764, 0.842) | 0.855 (0.781, 0.872) | p<0.001 |
| Areas of rainfed cropland per capita (m^2^), median (IQR) | 561.009 (393.191, 933.201) | 365.367 (198.156, 649.435) | 117.867 (94.356, 398.281) | p<0.001 |
| Areas of irrigated cropland per capita (m^2^), median (IQR) | 151.832 (113.764, 280.720) | 284.496 (175.330, 394.972) | 76.553 (41.832, 159.040) | p<0.001 |
| Areas of broad-leaved tree per capita (m^2^), median (IQR) | 348.562 (120.383, 583.732) | 400.098 (159.130, 1,231.380) | 1,551.577 (226.342, 5,306.656) | p<0.001 |
| Areas of needle-leaved tree per capita (m^2^), median (IQR) | 178.646 (87.693, 436.405) | 39.872 (16.643, 557.885) | 62.495 (21.442, 312.693) | 0.498 |
| Areas of shrubland per capita (m^2^), median (IQR) | 0.886 (0.338, 1.492) | 0.753 (0.030, 3.314) | 1.019 (0.593, 2.742) | 0.030 |
| Areas of grassland per capita (m^2^), median (IQR) | 2.021 (0.826, 5.149) | 4.744 (1.871, 26.427) | 19.608 (10.747, 35.783) | p<0.001 |
| Areas of wetland per capita (m^2^), median (IQR) | 23.361 (5.057, 53.619) | 4.067 (0.893, 10.802) | 10.097 (4.411, 59.910) | p<0.001 |
| Areas of built-up land per capita (m^2^), median (IQR) | 35.974 (27.070, 48.192) | 53.693 (39.339, 103.397) | 126.482 (89.216, 173.956) | p<0.001 |

HFRS: hemorrhagic fever with renal syndrome. IQR: interquartile range.

**Table S10. Monthly moving average of meteorological factors of each types I-III city during different lag periods.**

| **City** | **Province** | **Type** | **Temperature, median (IQR, ℃)** | | |  | **Rainfall, median (IQR, mm)** | | |
| --- | --- | --- | --- | --- | --- | --- | --- | --- | --- |
|  |  |  | **Lag=1** | **Lag=2** | **Lag=3** |  | **Lag=1** | **Lag=2** | **Lag=3** |
| Qiqihar | Heilongjiang | I | 9.179 (−10.288, 17.962) | 5.554 (−8.071, 18.162) | 7.759 (−8.125, 16.569) |  | 16.050 (6.231, 70.694) | 26.550 (6.833, 74.140) | 30.631 (8.494, 69.044) |
| Jixi | Heilongjiang | I | 8.725 (−8.962, 17.538) | 6.075 (−5.925, 16.812) | 8.175 (−6.686, 16.050) |  | 37.250 (18.512, 83.100) | 44.433 (19.375, 85.071) | 48.888 (20.612, 85.112) |
| Yichun | Heilongjiang | I | 7.825 (−12.450, 16.266) | 3.975 (−9.517, 16.192) | 6.462 (−10.200, 14.888) |  | 37.200 (12.438, 103.800) | 38.375 (13.758, 100.704) | 53.150 (16.288, 104.175) |
| Heihe | Heilongjiang | I | 7.002 (−13.956, 16.025) | 3.038 (−10.850, 16.265) | 5.300 (−11.306, 14.419) |  | 23.825 (8.844, 85.475) | 33.167 (10.650, 81.308) | 39.975 (11.388, 81.350) |
| Fushun | Liaoning | I | 10.475 (−5.362, 18.975) | 8.000 (−2.833, 18.746) | 10.225 (−3.599, 17.562) |  | 45.575 (20.700, 100.912) | 48.275 (21.604, 103.875) | 51.500 (24.188, 100.425) |
| Hulunbuir | Inner Mongolia | I | 5.233 (−15.412, 14.972) | 1.503 (−12.341, 15.112) | 3.917 (−12.411, 13.572) |  | 16.294 (5.753, 59.833) | 21.867 (6.274, 61.068) | 24.483 (7.339, 59.147) |
| Yantai | Shandong | I | 13.340 (4.155, 21.770) | 14.450 (4.415, 21.183) | 13.325 (5.364, 20.888) |  | 34.450 (17.610, 76.670) | 37.570 (19.385, 84.778) | 40.345 (21.930, 85.745) |
| Weifang | Shandong | I | 15.500 (4.575, 23.225) | 14.767 (6.108, 22.450) | 15.375 (5.825, 22.275) |  | 30.600 (14.650, 83.475) | 33.417 (15.225, 85.533) | 36.700 (17.250, 84.325) |
| Rizhao | Shandong | I | 14.600 (5.300, 22.317) | 14.817 (6.458, 21.696) | 14.725 (6.425, 21.778) |  | 43.175 (21.162, 92.750) | 47.367 (21.192, 102.358) | 54.050 (24.550, 104.650) |
| Linyi | Shandong | I | 16.125 (5.512, 23.513) | 15.372 (7.621, 22.850) | 16.638 (6.775, 22.462) |  | 46.375 (18.425, 107.363) | 49.675 (21.625, 116.412) | 56.800 (22.988, 126.637) |
| Zhumadian | Henan | I | 17.750 (7.475, 24.075) | 16.370 (9.383, 23.533) | 17.575 (8.425, 23.125) |  | 58.900 (30.225, 112.875) | 62.450 (32.875, 104.783) | 70.900 (36.950, 112.775) |
| Xi’an | Shaanxi | I | 16.104 (6.250, 23.500) | 15.417 (7.474, 23.078) | 16.675 (7.575, 22.700) |  | 39.400 (12.925, 72.075) | 44.050 (15.642, 72.658) | 45.450 (19.244, 68.275) |
| Baoji | Shaanxi | I | 15.125 (5.162, 21.486) | 13.792 (6.223, 21.212) | 14.988 (6.175, 20.912) |  | 49.750 (14.262, 82.762) | 49.242 (15.417, 86.587) | 47.150 (19.662, 87.488) |
| Xianyang | Shaanxi | I | 13.900 (3.474, 20.175) | 12.481 (5.192, 20.530) | 13.525 (4.771, 19.712) |  | 46.025 (13.150, 88.412) | 45.900 (16.602, 83.750) | 47.838 (20.700, 84.200) |
| Weinan | Shaanxi | I | 8.650 (−0.605, 14.550) | 7.483 (0.742, 14.725) | 8.875 (0.607, 13.800) |  | 54.750 (25.500, 100.175) | 59.250 (25.558, 98.175) | 62.075 (29.650, 98.400) |
| Lianyungang | Jiangsu | I | 14.900 (5.816, 22.815) | 15.202 (6.908, 22.400) | 15.400 (6.775, 22.325) |  | 48.700 (20.175, 117.375) | 53.633 (24.675, 123.475) | 61.650 (28.450, 125.950) |
| Yancheng | Jiangsu | I | 15.250 (7.175, 23.100) | 16.450 (8.208, 23.213) | 16.025 (8.162, 22.650) |  | 63.525 (36.962, 126.825) | 67.025 (40.254, 130.900) | 75.562 (44.262, 124.125) |
| Taizhou | Zhejiang | I | 18.600 (11.400, 25.408) | 19.256 (11.928, 24.997) | 18.450 (12.516, 24.567) |  | 117.183 (75.042, 178.750) | 124.772 (78.692, 180.613) | 130.192 (84.542, 168.700) |
| Daqing | Heilongjiang | II | 9.650 (−8.850, 18.600) | 6.433 (−6.875, 18.842) | 8.825 (−6.700, 17.095) |  | 20.200 (6.525, 67.025) | 24.533 (7.725, 67.925) | 33.000 (10.525, 75.325) |
| Changchun | Jilin | II | 10.150 (−6.175, 19.225) | 7.933 (−4.767, 19.042) | 9.750 (−4.075, 17.750) |  | 36.100 (13.075, 96.075) | 36.533 (13.658, 91.408) | 43.900 (16.950, 89.750) |
| Jilin | Jilin | II | 9.400 (-7.316, 18.055) | 6.901 (-5.410, 17.908) | 9.050 (-5.608, 16.675) |  | 41.342 (16.700, 109.288) | 45.522 (19.961, 107.603) | 54.517 (23.692, 104.246) |
| Siping | Jilin | II | 10.800 (−5.888, 19.388) | 8.227 (−3.496, 19.371) | 10.500 (−3.475, 18.000) |  | 27.425 (10.250, 75.050) | 31.908 (11.967, 78.375) | 39.012 (13.900, 79.000) |
| Baicheng | Jilin | II | 10.150 (−7.350, 19.287) | 7.196 (−5.133, 19.277) | 9.815 (−4.863, 17.762) |  | 18.300 (3.412, 61.025) | 26.883 (5.312, 62.487) | 28.975 (8.591, 60.900) |
| Jinzhou | Liaoning | II | 11.789 (−2.081, 20.597) | 10.590 (0.002, 20.222) | 12.072 (−0.196, 19.386) |  | 32.728 (9.444, 77.000) | 35.583 (12.200, 81.192) | 39.489 (14.989, 83.139) |
| Yingkou | Liaoning | II | 12.025 (−0.462, 20.943) | 11.300 (1.221, 20.374) | 12.225 (0.913, 19.962) |  | 35.000 (12.938, 78.925) | 38.683 (13.887, 85.912) | 42.088 (17.188, 90.788) |
| Chaoyang | Liaoning | II | 12.650 (−2.013, 20.912) | 10.583 (0.200, 20.709) | 12.112 (−0.088, 19.491) |  | 23.625 (5.525, 64.112) | 25.117 (6.225, 65.538) | 29.312 (10.112, 65.162) |
| Huludao | Liaoning | II | 11.375 (−0.950, 20.737) | 10.950 (1.312, 19.746) | 12.012 (0.850, 19.425) |  | 27.750 (5.750, 66.075) | 27.667 (7.525, 70.342) | 32.700 (9.913, 87.712) |
| Tangshan | Hebei | II | 14.950 (1.525, 22.558) | 12.961 (3.906, 21.861) | 14.600 (3.433, 21.383) |  | 27.417 (7.200, 76.758) | 34.356 (9.578, 87.272) | 32.575 (11.708, 91.400) |
| Qinhuangdao | Hebei | II | 12.925 (−0.137, 20.776) | 11.342 (2.046, 20.517) | 12.500 (1.426, 19.612) |  | 23.000 (5.350, 69.038) | 30.408 (7.392, 82.967) | 31.675 (10.075, 88.962) |
| Jining | Shandong | II | 16.450 (4.875, 23.600) | 14.950 (7.142, 23.150) | 16.500 (6.150, 22.450) |  | 33.700 (13.400, 80.325) | 39.633 (17.058, 95.067) | 41.125 (18.200, 96.775) |
| Fuzhou | Fujian | II | 20.975 (15.375, 26.425) | 21.542 (14.846, 26.137) | 20.612 (15.788, 25.975) |  | 111.475 (57.150, 168.150) | 115.542 (64.321, 166.171) | 114.675 (71.362, 175.337) |
| Quanzhou | Fujian | II | 17.200 (11.944, 21.675) | 17.475 (12.120, 21.637) | 17.062 (12.675, 21.150) |  | 119.550 (55.900, 179.687) | 120.233 (69.433, 176.246) | 122.275 (71.612, 171.425) |
| Nanping | Fujian | II | 19.120 (12.206, 25.440) | 19.860 (13.447, 25.225) | 19.498 (12.990, 24.745) |  | 146.120 (75.325, 226.600) | 148.990 (84.535, 215.012) | 153.085 (95.435, 228.900) |
| Ningde | Fujian | II | 20.275 (13.688, 26.000) | 20.683 (14.108, 25.958) | 19.988 (14.650, 25.575) |  | 151.875 (91.550, 212.462) | 153.150 (94.825, 232.696) | 163.588 (101.638, 228.738) |
| Shaoyang | Hunan | II | 18.240 (10.809, 25.128) | 18.338 (12.126, 24.637) | 18.618 (11.640, 24.039) |  | 114.625 (70.470, 189.998) | 121.777 (79.878, 178.388) | 125.935 (83.110, 175.137) |
| Liangshan | Sichuan | II | 15.743 (9.763, 19.379) | 15.193 (10.213, 19.108) | 15.693 (10.668, 18.718) |  | 52.265 (11.914, 144.336) | 65.714 (17.612, 145.349) | 70.414 (20.661, 141.793) |
| Guangzhou | Guangdong | II | 22.950 (17.400, 27.312) | 23.065 (17.875, 27.027) | 22.925 (17.888, 26.500) |  | 147.800 (63.375, 289.600) | 170.442 (69.671, 288.354) | 176.212 (83.688, 284.100) |
| Shenzhen | Guangdong | II | 24.250 (19.300, 27.950) | 24.500 (19.336, 27.658) | 23.775 (19.600, 27.275) |  | 102.300 (34.250, 225.350) | 113.450 (39.875, 226.192) | 126.525 (48.575, 225.200) |
| Foshan | Guangdong | II | 22.694 (17.147, 27.478) | 23.157 (17.572, 27.077) | 22.708 (17.767, 26.609) |  | 145.128 (66.647, 270.759) | 167.211 (73.687, 250.385) | 173.872 (86.450, 252.683) |
| Chuxiong | Yunnan | II | 18.618 (13.230, 21.551) | 17.965 (13.510, 21.511) | 18.270 (13.986, 21.084) |  | 57.562 (18.415, 148.330) | 61.927 (22.052, 140.306) | 67.612 (25.590, 142.314) |
| Dali | Yunnan | II | 16.550 (12.274, 19.625) | 16.417 (12.283, 19.458) | 16.300 (12.600, 19.125) |  | 56.050 (18.500, 142.050) | 62.633 (22.242, 135.283) | 67.775 (25.350, 133.175) |
| Harbin | Heilongjiang | III | 9.038 (-9.606, 17.862) | 5.771 (-6.892, 17.769) | 8.169 (-7.870, 16.319) |  | 31.475 (14.094, 75.675) | 37.558 (13.460, 80.519) | 43.994 (16.219, 81.825) |
| Hegang | Heilongjiang | III | 7.750 (-11.046, 16.675) | 4.450 (-8.125, 15.762) | 6.325 (-8.175, 14.920) |  | 39.400 (12.025, 104.875) | 45.450 (13.842, 101.850) | 53.675 (16.550, 100.300) |
| Shuangyashan | Heilongjiang | III | 9.300 (-8.925, 18.025) | 6.383 (-6.217, 17.483) | 8.400 (-6.750, 16.400) |  | 32.600 (11.800, 79.575) | 37.483 (13.125, 83.467) | 41.775 (14.725, 79.650) |
| Jiamusi | Heilongjiang | III | 8.975 (-10.462, 17.638) | 5.575 (-7.710, 17.267) | 7.825 (-8.300, 15.850) |  | 33.550 (12.637, 83.612) | 42.967 (13.742, 82.933) | 47.262 (15.400, 82.650) |
| Mudanjiang | Heilongjiang | III | 8.175 (-8.400, 16.588) | 5.767 (-5.808, 16.212) | 7.638 (-6.475, 15.238) |  | 40.750 (15.925, 86.600) | 46.083 (17.833, 91.742) | 49.412 (21.625, 87.262) |
| Suihua | Heilongjiang | III | 8.662 (-11.044, 17.722) | 5.305 (-8.438, 17.710) | 7.481 (-8.425, 16.062) |  | 25.912 (7.812, 87.006) | 31.383 (9.690, 86.829) | 38.775 (10.138, 86.375) |
| Tonghua | Jilin | III | 10.475 (-4.412, 18.788) | 8.467 (-2.079, 18.392) | 10.250 (-3.062, 17.405) |  | 56.825 (24.712, 105.975) | 54.508 (28.704, 115.592) | 61.000 (30.950, 116.388) |
| Baishan | Jilin | III | 7.600 (-8.200, 16.083) | 5.389 (-5.694, 15.643) | 7.275 (-6.217, 14.658) |  | 47.150 (22.842, 97.242) | 51.872 (24.492, 99.258) | 55.283 (28.092, 97.425) |
| Yanbian | Jilin | III | 8.370 (-7.390, 16.795) | 6.130 (-5.023, 16.135) | 7.830 (-5.395, 15.330) |  | 40.000 (14.970, 82.220) | 41.207 (16.583, 89.298) | 43.960 (18.565, 88.970) |
| Shenyang | Liaoning | III | 11.525 (-3.299, 20.375) | 9.797 (-1.237, 19.942) | 11.712 (-1.575, 19.050) |  | 33.775 (13.275, 78.575) | 40.225 (14.942, 81.700) | 44.138 (18.250, 85.525) |
| Benxi | Liaoning | III | 10.325 (-3.538, 19.388) | 9.083 (-1.537, 18.819) | 10.638 (-1.625, 17.975) |  | 43.775 (17.725, 105.350) | 49.392 (21.404, 111.696) | 58.850 (24.238, 114.700) |
| Dandong | Liaoning | III | 10.000 (-3.199, 18.725) | 8.750 (-1.275, 18.433) | 10.175 (-1.752, 17.450) |  | 53.550 (22.800, 128.500) | 58.033 (28.308, 150.258) | 69.700 (29.450, 152.350) |
| Tieling | Liaoning | III | 11.200 (-4.450, 19.870) | 9.033 (-2.533, 19.933) | 11.175 (-2.575, 18.566) |  | 35.850 (15.750, 90.300) | 39.400 (17.308, 92.275) | 48.150 (19.525, 96.600) |
| Jinan | Shandong | III | 17.450 (5.725, 24.075) | 15.833 (7.325, 23.675) | 17.375 (7.325, 23.075) |  | 32.350 (14.925, 79.150) | 30.833 (16.025, 99.383) | 38.850 (19.475, 99.075) |
| Qingdao | Shandong | III | 13.750 (5.137, 21.184) | 15.433 (5.537, 21.450) | 13.475 (6.775, 21.200) |  | 41.900 (16.850, 70.550) | 42.617 (20.467, 75.658) | 43.275 (23.100, 85.225) |
| Zibo | Shandong | III | 16.033 (4.567, 23.300) | 14.683 (6.289, 22.635) | 16.475 (5.892, 22.183) |  | 35.383 (14.975, 85.642) | 38.400 (15.192, 93.786) | 43.208 (18.300, 101.325) |
| Ningbo | Zhejiang | III | 17.975 (10.350, 25.272) | 18.925 (10.804, 25.089) | 18.188 (11.387, 24.612) |  | 113.300 (70.738, 177.637) | 117.175 (83.112, 176.387) | 127.512 (85.050, 169.812) |
| Shaoxing | Zhejiang | III | 17.550 (9.875, 24.906) | 18.617 (10.983, 24.633) | 18.025 (10.905, 24.075) |  | 108.950 (72.050, 165.475) | 123.350 (78.508, 165.517) | 122.825 (83.075, 159.925) |
| Quzhou | Zhejiang | III | 18.064 (10.629, 24.930) | 18.877 (12.042, 24.612) | 18.577 (11.571, 24.185) |  | 147.964 (77.207, 223.866) | 146.180 (89.569, 222.657) | 146.657 (100.825, 219.004) |
| Lishui | Zhejiang | III | 19.125 (11.795, 25.775) | 19.808 (13.250, 25.579) | 19.549 (12.662, 25.072) |  | 125.875 (77.062, 184.262) | 132.333 (85.579, 184.367) | 129.775 (87.075, 183.912) |
| Nanchang | Jiangxi | III | 19.250 (11.150, 26.550) | 19.812 (12.457, 26.108) | 20.075 (12.175, 25.704) |  | 119.100 (70.600, 209.850) | 141.850 (72.375, 209.208) | 140.750 (81.575, 205.700) |
| Yichun | Jiangxi | III | 19.250 (11.100, 26.295) | 19.217 (12.637, 25.762) | 19.787 (12.050, 25.175) |  | 133.000 (83.488, 214.225) | 139.933 (88.404, 204.804) | 136.550 (96.962, 197.312) |
| Fuzhou | Jiangxi | III | 19.525 (11.500, 25.962) | 19.802 (13.308, 25.846) | 19.930 (12.612, 25.125) |  | 137.050 (78.075, 230.050) | 144.883 (81.892, 215.408) | 146.438 (88.950, 219.138) |
| Shangrao | Jiangxi | III | 18.800 (10.960, 25.536) | 19.292 (12.329, 25.725) | 19.612 (12.000, 24.988) |  | 129.825 (74.113, 227.562) | 137.750 (80.117, 227.662) | 148.648 (88.425, 217.075) |
| Xiangyang | Hubei | III | 18.100 (8.238, 24.500) | 17.033 (10.483, 24.004) | 18.162 (9.312, 23.750) |  | 57.175 (27.600, 91.187) | 62.142 (32.354, 89.800) | 60.538 (33.675, 87.288) |
| Jingmen | Hubei | III | 18.050 (9.300, 24.975) | 17.817 (10.914, 24.593) | 18.745 (10.150, 24.082) |  | 67.900 (35.000, 112.150) | 68.817 (37.642, 110.525) | 73.000 (43.200, 110.050) |
| Jingzhou | Hubei | III | 18.200 (9.558, 24.885) | 17.783 (10.842, 24.750) | 18.950 (10.450, 24.100) |  | 87.150 (43.075, 130.175) | 94.633 (47.292, 128.717) | 95.400 (50.125, 125.250) |
| Changsha | Hunan | III | 19.025 (10.525, 25.587) | 18.500 (11.954, 24.996) | 19.123 (11.375, 25.000) |  | 110.175 (66.700, 162.362) | 113.417 (73.096, 152.587) | 110.988 (81.125, 151.725) |
| Xiangtan | Hunan | III | 18.054 (10.279, 24.681) | 18.198 (11.504, 24.601) | 18.617 (11.011, 23.971) |  | 117.950 (72.043, 164.571) | 120.190 (80.877, 165.675) | 122.246 (86.318, 161.504) |
| Hengyang | Hunan | III | 15.917 (9.562, 23.112) | 16.983 (9.416, 23.083) | 16.225 (10.433, 22.817) |  | 125.583 (79.792, 180.342) | 126.050 (84.694, 168.700) | 126.383 (92.425, 166.767) |
| Yiyang | Hunan | III | 18.267 (10.417, 25.017) | 18.267 (11.444, 24.774) | 18.958 (11.108, 24.433) |  | 112.267 (71.733, 168.475) | 117.911 (74.931, 162.514) | 117.483 (79.133, 160.067) |
| Chenzhou | Hunan | III | 19.800 (11.375, 25.824) | 19.417 (12.375, 25.496) | 19.600 (12.400, 24.925) |  | 119.900 (72.725, 170.725) | 122.533 (80.583, 162.900) | 124.500 (86.950, 166.350) |
| Huaihua | Hunan | III | 17.517 (10.350, 24.633) | 17.756 (11.567, 23.894) | 18.150 (11.133, 23.575) |  | 104.817 (62.917, 178.975) | 108.150 (69.989, 171.183) | 115.692 (73.408, 174.983) |
| Loudi | Hunan | III | 18.350 (10.425, 25.325) | 18.267 (11.831, 25.222) | 18.850 (11.375, 24.700) |  | 104.800 (64.525, 150.525) | 104.383 (71.158, 153.010) | 109.450 (77.200, 157.000) |
| Zunyi | Guizhou | III | 16.050 (9.150, 22.262) | 16.195 (9.987, 21.619) | 16.612 (10.051, 21.500) |  | 75.950 (36.812, 128.900) | 83.717 (40.854, 126.146) | 81.800 (46.237, 120.050) |

**Table S10 (Continued).**

| **City** | **Province** | **Type** | **RH, median (IQR, %)** | | |
| --- | --- | --- | --- | --- | --- |
|  |  |  | **Lag=1** | **Lag=2** | **Lag=3** |
| Qiqihar | Heilongjiang | I | 62.688 (56.329, 68.208) | 62.968 (55.654, 66.902) | 62.432 (56.773, 66.900) |
| Jixi | Heilongjiang | I | 65.146 (59.902, 72.810) | 65.747 (60.216, 72.279) | 66.211 (60.871, 72.136) |
| Yichun | Heilongjiang | I | 69.004 (64.219, 75.539) | 68.922 (64.332, 74.219) | 69.262 (64.752, 74.557) |
| Heihe | Heilongjiang | I | 66.580 (62.053, 72.561) | 67.095 (62.290, 71.494) | 67.104 (61.870, 71.367) |
| Fushun | Liaoning | I | 67.314 (60.228, 73.530) | 67.474 (61.070, 73.122) | 67.317 (61.442, 72.930) |
| Hulunbuir | Inner Mongolia | I | 63.618 (57.806, 67.926) | 63.934 (57.508, 67.040) | 63.498 (57.149, 66.525) |
| Yantai | Shandong | I | 63.713 (59.552, 70.998) | 64.538 (59.908, 70.535) | 64.476 (60.862, 69.990) |
| Weifang | Shandong | I | 63.563 (57.441, 70.953) | 63.782 (58.214, 70.408) | 63.986 (58.454, 70.021) |
| Rizhao | Shandong | I | 67.340 (62.038, 75.391) | 67.338 (62.348, 75.480) | 67.317 (63.243, 74.497) |
| Linyi | Shandong | I | 66.545 (60.396, 75.111) | 67.221 (61.031, 74.505) | 67.477 (61.886, 73.357) |
| Zhumadian | Henan | I | 69.357 (64.742, 74.382) | 69.275 (65.197, 73.665) | 69.602 (65.944, 74.045) |
| Xi’an | Shaanxi | I | 64.432 (59.732, 72.177) | 64.486 (59.561, 71.127) | 65.127 (60.188, 70.086) |
| Baoji | Shaanxi | I | 67.533 (62.106, 75.701) | 67.279 (61.986, 74.959) | 67.640 (62.529, 74.301) |
| Xianyang | Shaanxi | I | 64.821 (59.530, 73.490) | 65.142 (59.417, 72.288) | 65.515 (60.413, 71.002) |
| Weinan | Shaanxi | I | 61.902 (56.648, 70.324) | 61.813 (56.907, 69.099) | 62.550 (57.424, 69.169) |
| Lianyungang | Jiangsu | I | 71.187 (66.944, 77.669) | 71.617 (66.956, 78.251) | 71.536 (67.470, 77.411) |
| Yancheng | Jiangsu | I | 75.833 (71.640, 79.686) | 75.719 (71.881, 79.446) | 75.500 (72.109, 79.437) |
| Taizhou | Zhejiang | I | 76.692 (73.191, 80.305) | 76.352 (73.888, 79.794) | 76.682 (73.782, 79.754) |
| Daqing | Heilongjiang | II | 63.685 (56.644, 70.439) | 64.227 (57.239, 69.036) | 63.669 (58.437, 68.838) |
| Changchun | Jilin | II | 64.162 (57.792, 71.365) | 65.224 (58.102, 70.967) | 65.099 (59.562, 69.774) |
| Jilin | Jilin | II | 67.683 (62.187, 74.271) | 67.781 (62.225, 73.559) | 67.547 (63.184, 72.621) |
| Siping | Jilin | II | 63.545 (56.497, 70.891) | 64.009 (56.489, 70.217) | 63.791 (57.149, 69.827) |
| Baicheng | Jilin | II | 55.966 (48.463, 65.682) | 56.791 (49.966, 63.709) | 56.657 (50.945, 62.934) |
| Jinzhou | Liaoning | II | 57.694 (49.769, 67.036) | 57.296 (50.110, 67.042) | 58.119 (50.960, 65.956) |
| Yingkou | Liaoning | II | 64.328 (57.475, 71.812) | 64.293 (58.171, 71.334) | 64.170 (58.586, 70.958) |
| Chaoyang | Liaoning | II | 51.819 (42.700, 63.325) | 52.373 (43.213, 63.228) | 52.433 (44.932, 61.685) |
| Huludao | Liaoning | II | 58.063 (50.347, 69.875) | 58.389 (51.400, 69.374) | 59.936 (52.103, 67.673) |
| Tangshan | Hebei | II | 59.799 (52.437, 69.706) | 60.357 (53.819, 68.616) | 60.529 (54.041, 68.386) |
| Qinhuangdao | Hebei | II | 62.022 (55.132, 72.999) | 62.170 (55.993, 72.684) | 62.623 (56.814, 71.254) |
| Jining | Shandong | II | 67.787 (61.865, 75.030) | 68.353 (62.058, 74.566) | 68.314 (62.694, 74.229) |
| Fuzhou | Fujian | II | 76.147 (72.740, 78.967) | 76.519 (73.058, 79.315) | 76.437 (72.648, 78.901) |
| Quanzhou | Fujian | II | 78.336 (73.761, 81.677) | 78.268 (74.112, 81.500) | 77.698 (73.853, 81.394) |
| Nanping | Fujian | II | 78.361 (76.293, 81.540) | 78.523 (76.534, 81.038) | 78.706 (76.350, 81.021) |
| Ningde | Fujian | II | 78.068 (75.036, 80.818) | 78.134 (75.353, 80.317) | 78.187 (75.434, 80.179) |
| Shaoyang | Hunan | II | 77.379 (74.633, 81.034) | 77.247 (73.961, 80.919) | 76.844 (74.116, 81.149) |
| Liangshan | Sichuan | II | 68.611 (59.928, 75.580) | 69.277 (60.744, 75.144) | 67.845 (60.411, 73.990) |
| Guangzhou | Guangdong | II | 78.573 (73.494, 82.047) | 77.968 (74.084, 81.737) | 78.170 (74.284, 81.857) |
| Shenzhen | Guangdong | II | 76.579 (71.880, 80.167) | 75.903 (71.869, 80.103) | 76.183 (71.965, 79.731) |
| Foshan | Guangdong | II | 78.429 (73.198, 81.501) | 78.301 (73.381, 81.703) | 77.922 (73.729, 81.513) |
| Chuxiong | Yunnan | II | 66.671 (53.413, 73.928) | 64.987 (53.974, 73.654) | 64.881 (55.556, 72.782) |
| Dali | Yunnan | II | 65.263 (55.633, 77.041) | 66.489 (56.229, 75.281) | 66.408 (57.126, 74.877) |
| Harbin | Heilongjiang | III | 68.156 (62.066, 74.577) | 68.865 (63.142, 73.237) | 68.596 (63.430, 73.287) |
| Hegang | Heilongjiang | III | 68.136 (63.512, 74.769) | 67.583 (63.994, 73.565) | 68.325 (64.517, 73.682) |
| Shuangyashan | Heilongjiang | III | 67.248 (62.070, 74.679) | 67.190 (63.008, 73.444) | 67.857 (63.931, 73.101) |
| Jiamusi | Heilongjiang | III | 67.297 (62.586, 74.533) | 66.982 (63.647, 73.295) | 67.953 (64.043, 73.304) |
| Mudanjiang | Heilongjiang | III | 65.467 (59.533, 73.030) | 65.299 (59.525, 71.870) | 65.182 (60.974, 72.364) |
| Suihua | Heilongjiang | III | 66.892 (61.009, 73.472) | 67.318 (61.875, 71.564) | 66.747 (62.305, 70.991) |
| Tonghua | Jilin | III | 67.624 (61.381, 74.392) | 68.153 (61.431, 73.589) | 67.724 (62.435, 73.690) |
| Baishan | Jilin | III | 67.554 (61.897, 74.930) | 68.369 (62.539, 74.187) | 68.031 (63.092, 73.897) |
| Yanbian | Jilin | III | 64.054 (57.686, 73.274) | 64.037 (57.981, 72.811) | 64.082 (58.630, 72.159) |
| Shenyang | Liaoning | III | 63.242 (55.121, 70.430) | 63.044 (56.135, 69.864) | 63.281 (56.552, 69.762) |
| Benxi | Liaoning | III | 64.219 (57.017, 71.351) | 64.762 (57.580, 70.832) | 64.388 (58.715, 70.153) |
| Dandong | Liaoning | III | 66.084 (60.736, 75.292) | 67.041 (60.850, 76.372) | 66.827 (61.975, 74.884) |
| Tieling | Liaoning | III | 64.292 (57.005, 71.131) | 65.012 (57.507, 71.553) | 64.763 (58.535, 70.505) |
| Jinan | Shandong | III | 57.738 (51.343, 67.443) | 58.921 (51.980, 67.319) | 59.311 (52.583, 66.570) |
| Qingdao | Shandong | III | 66.385 (61.814, 74.467) | 66.829 (62.407, 73.301) | 67.261 (63.387, 73.528) |
| Zibo | Shandong | III | 60.209 (52.415, 68.893) | 60.497 (53.545, 68.925) | 60.303 (54.232, 68.584) |
| Ningbo | Zhejiang | III | 75.919 (73.094, 79.080) | 76.001 (73.576, 78.818) | 75.880 (73.371, 78.726) |
| Shaoxing | Zhejiang | III | 75.349 (71.850, 77.747) | 74.740 (72.691, 77.158) | 74.901 (72.691, 76.866) |
| Quzhou | Zhejiang | III | 75.620 (72.753, 78.624) | 75.624 (73.067, 77.865) | 75.436 (73.369, 78.048) |
| Lishui | Zhejiang | III | 75.525 (73.265, 78.191) | 75.529 (73.479, 77.909) | 75.596 (73.561, 77.858) |
| Nanchang | Jiangxi | III | 74.413 (70.843, 78.447) | 74.600 (71.300, 77.927) | 74.725 (71.337, 77.622) |
| Yichun | Jiangxi | III | 78.139 (75.256, 81.313) | 78.378 (75.542, 80.699) | 78.467 (75.667, 80.348) |
| Fuzhou | Jiangxi | III | 77.527 (74.728, 80.714) | 77.666 (75.212, 80.254) | 77.440 (75.299, 80.161) |
| Shangrao | Jiangxi | III | 76.249 (73.937, 79.994) | 76.495 (74.071, 79.410) | 76.672 (73.805, 79.034) |
| Xiangyang | Hubei | III | 73.152 (69.487, 77.122) | 72.965 (70.086, 76.897) | 73.122 (70.238, 76.156) |
| Jingmen | Hubei | III | 75.026 (72.230, 77.999) | 74.719 (72.333, 77.809) | 74.905 (72.541, 77.519) |
| Jingzhou | Hubei | III | 77.088 (74.205, 79.643) | 76.992 (74.371, 79.136) | 77.082 (74.160, 78.904) |
| Changsha | Hunan | III | 77.035 (73.273, 80.381) | 77.682 (73.652, 79.977) | 76.559 (73.656, 80.216) |
| Xiangtan | Hunan | III | 77.072 (73.588, 81.147) | 77.532 (73.436, 80.610) | 77.100 (73.833, 80.548) |
| Hengyang | Hunan | III | 75.952 (73.571, 80.074) | 76.819 (72.955, 79.266) | 76.780 (73.295, 79.565) |
| Yiyang | Hunan | III | 76.639 (72.903, 80.239) | 76.425 (73.145, 80.411) | 76.269 (72.938, 80.445) |
| Chenzhou | Hunan | III | 77.294 (73.114, 80.305) | 76.913 (73.392, 80.228) | 77.103 (73.082, 80.511) |
| Huaihua | Hunan | III | 78.742 (75.734, 82.201) | 78.567 (75.676, 81.852) | 78.551 (75.791, 81.900) |
| Loudi | Hunan | III | 76.575 (73.332, 80.248) | 76.698 (73.294, 79.811) | 76.430 (73.490, 80.133) |
| Zunyi | Guizhou | III | 78.883 (76.291, 81.679) | 78.783 (76.743, 81.226) | 79.000 (76.961, 81.046) |

**Fig. S4. Pooled exposure-response in relative risks between the meteorological factors and the monthly incidence rates of HFRS during different lag periods in Type I cities.** The red lines indicate the pooled exposure-response with its 95%CI in shaded area, and the gray dashed lines indicate the exposure-response of each Type I city. The parameter estimations of three monthly meteorological factors including average temperature, cumulative rainfall and average RH were all transformed into percentile scale in analysis with the references at their respective medians, while the SPI retained its original scale with the reference at 0. The time unit of lag period is month.

HFRS: hemorrhagic fever with renal syndrome. CI: confidence interval. SPI: standardized precipitation index.

**
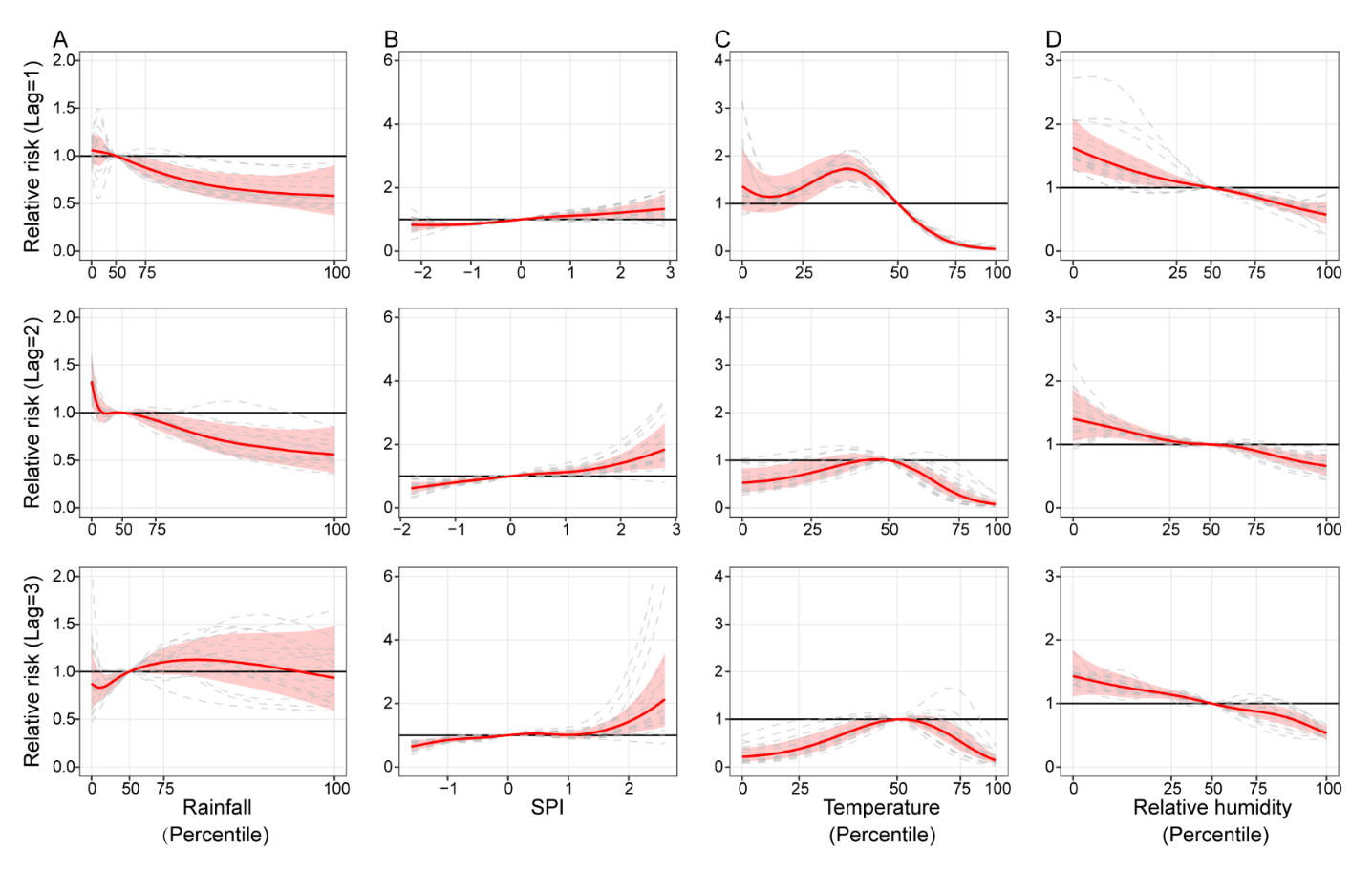
**

**Fig. S5. Pooled exposure-response in relative risks between the meteorological factors and the monthly incidence rates of HFRS during different lag periods in Type II cities.** The red lines indicate the pooled exposure-response with its 95%CI in shaded area, and the gray dashed lines indicate the exposure-response of each Type II city. The parameter estimations of three monthly meteorological factors including average temperature, cumulative rainfall and average RH were all transformed into percentile scale in analysis with the references at their respective medians, while the SPI retained its original scale with the reference at 0. The time unit of lag period is month.

HFRS: hemorrhagic fever with renal syndrome. CI: confidence interval. SPI: standardized precipitation index.

**
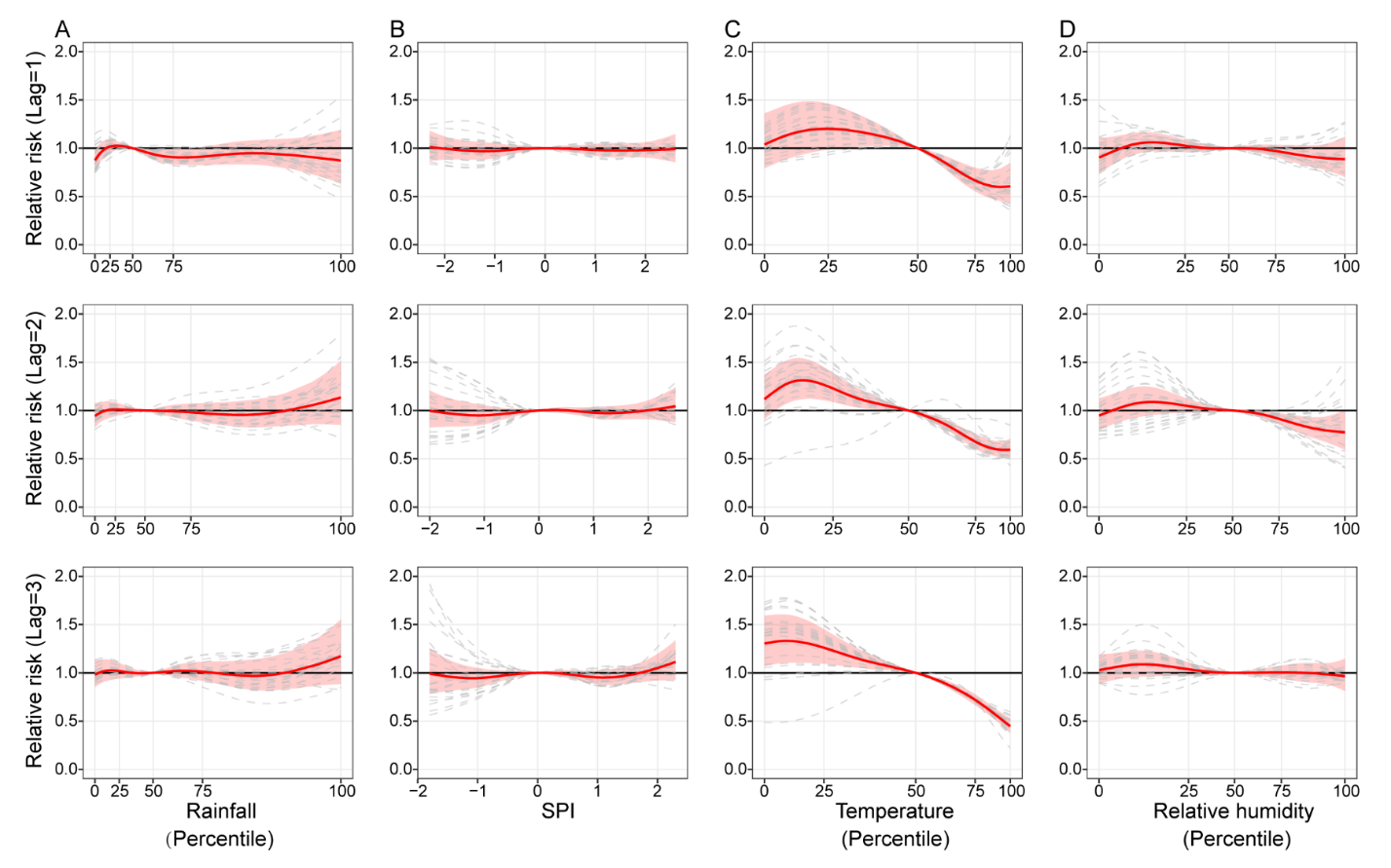
**

**Fig. S6. Pooled exposure-response in relative risks between the meteorological factors and the monthly incidence rates of HFRS during different lag periods in Type III cities.** The red lines indicate the pooled exposure-response with its 95%CI in shaded area, and the gray dashed lines indicate the exposure-response of each Type III city. The parameter estimations of three monthly meteorological factors including average temperature, cumulative rainfall and average RH were all transformed into percentile scale in analysis with the references at their respective medians, while the SPI retained its original scale with the reference at 0. The time unit of lag period is month.

HFRS: hemorrhagic fever with renal syndrome. CI: confidence interval. SPI: standardized precipitation index.

**
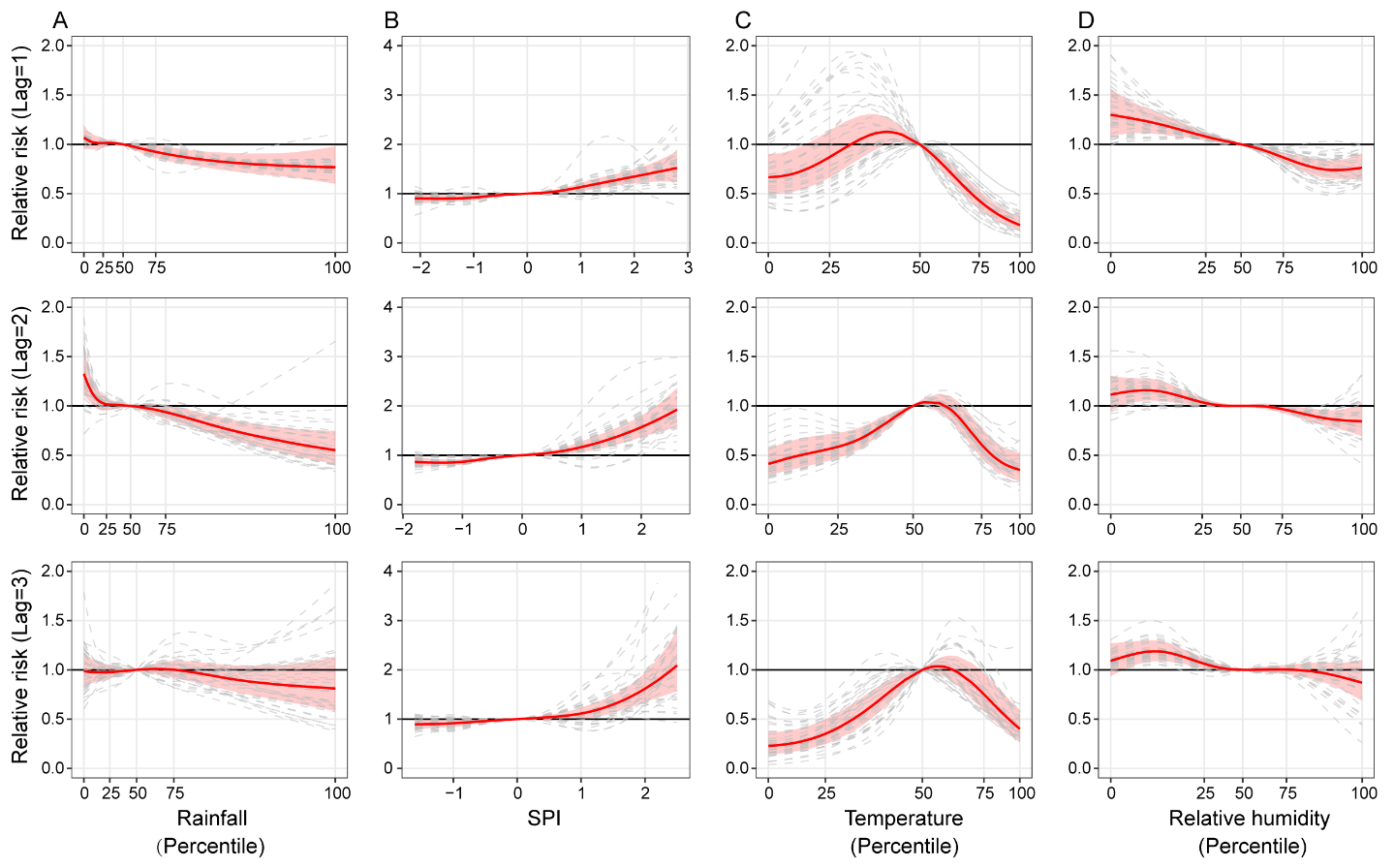
**
